# Unraveling the Molecular Signatures of Oxidative Phosphorylation to Cope with the Nutritionally Changing Metabolic Capabilities of Liver and Muscle Tissues in Farmed Fish

**DOI:** 10.1371/journal.pone.0122889

**Published:** 2015-04-15

**Authors:** Azucena Bermejo-Nogales, Josep Alvar Calduch-Giner, Jaume Pérez-Sánchez

**Affiliations:** Nutrigenomics and Fish Growth Endocrinology Group, Institute of Aquaculture Torre de la Sal (CSIC-IATS), Ribera de Cabanes, Castellón, Spain; Ben-Gurion University of the Negev, ISRAEL

## Abstract

Mitochondrial oxidative phosphorylation provides over 90% of the energy produced by aerobic organisms, therefore the regulation of mitochondrial activity is a major issue for coping with the changing environment and energy needs. In fish, there is a large body of evidence of adaptive changes in enzymatic activities of the OXPHOS pathway, but less is known at the transcriptional level and the first aim of the present study was to define the molecular identity of the actively transcribed subunits of the mitochondrial respiratory chain of a livestock animal, using gilthead sea bream as a model of farmed fish with a high added value for European aquaculture. Extensive BLAST searches in our transcriptomic database (www.nutrigroup-iats.org/seabreamdb) yielded 97 new sequences with a high coverage of catalytic, regulatory and assembly factors of Complex I to V. This was the basis for the development of a PCR array for the simultaneous profiling of 88 selected genes. This new genomic resource allowed the differential gene expression of liver and muscle tissues in a model of 10 fasting days. A consistent down-regulated response involving 72 genes was made by the liver, whereas an up-regulated response with 29 and 10 differentially expressed genes was found in white skeletal muscle and heart, respectively. This differential regulation was mostly mediated by nuclear-encoded genes (skeletal muscle) or both mitochondrial- and nuclear-encoded genes (liver, heart), which is indicative of a complex and differential regulation of mitochondrial and nuclear genomes, according to the changes in the lipogenic activity of liver and the oxidative capacity of glycolytic and highly oxidative muscle tissues. These insights contribute to the identification of the most responsive elements of OXPHOS in each tissue, which is of relevance for the appropriate gene targeting of nutritional and/or environmental metabolic disturbances in livestock animals.

## Introduction

The main cellular function of mitochondria is the production of ATP by oxidation of metabolic fuels in the tricarboxylic acid (TCA) cycle and oxidative phosphorylation (OXPHOS) pathway. In this process, NADH and FADH_2_ function as electron donors of Complex I (NADH: ubiquinone oxidoreductase) and Complex II (succinate dehydrogenase) that are transported through Complex III (ubiquinol cytochrome c reductase) to Complex IV (cytochrome c oxidase), where molecular oxygen serves as the final electron acceptor of the mitochondrial respiratory chain. This electron transport generates a proton gradient across the inner-mitochondrial membrane coupled with Complex V (ATP synthase) to the synthesis of ATP from ADP and P_i_. In mammals, this process is highly regulated at the transcriptional level, with the mitochondrial translation machinery becoming responsible of the synthesis of 13 catalytic and highly hydrophobic proteins of the mitochondrial respiratory chain. However, more than 70 OXPHOS proteins are encoded by nuclear DNA (nDNA), imported from the cytosol, and translocated across outer and inner mitochondrial membranes by conserved molecular chaperones and protein components of the TOM/TIM complex [1,2]. All this, therefore, is encompassed by a complex regulation of nuclear and mitochondrial genomes, which involves hundreds of genes controlling the expression, function, transport, assembly and turnover of mitochondrial proteins and enzyme subunits of OXPHOS in particular [3].

Attempts to assess the wide gene expression regulation of OXPHOS by fasting and caloric restriction have been addressed in humans and other experimental models of mammals. Importantly, the achieved response depends on the tissue and intensity of nutritional stress stimuli, but a common rule is the down-regulation of OXPHOS in adipose tissue and liver, which in turn is followed by the up-regulation of OXPHOS in skeletal muscle [4]. In fish, there is a large body of evidence of adaptive changes in enzyme activities of OXPHOS with changes in metabolic capabilities [5,6], diet composition [7,8], thermal condition [9–11] and exposure to environmental pollutants [12,13]. Less is known at the molecular level, but this situation is changing with the advent of wide gene expression analysis, and more and more information is coming from the transcriptionally mediated effects of hypoxia, pollutants and environmental conditions upon the OXPHOS of a wide range of fish species, including fish species models [14,15] and wild/farmed fish, such as European eel [16,17], salmon [18] and trout [19]. Experimental data also reveal a relatively high conservation of OXPHOS enzymes in the genome of teleostean fish lineages [20]. However, the molecular identity and, more importantly, the transcriptional plasticity of OXPHOS in a given tissue and/or fish species remain mostly unexplored.

In gilthead sea bream, a highly cultured fish in the whole of the Mediterranean area, attempts to phenotype the transcriptionally mediated response of hepatic mitochondria under acute and chronic stress have been proved highly informative to underline the health and welfare of farmed fish [21,22]. In addition, meta-analysis of microarray data using the on-line Fish and Chip tool (www.fishandchips.genouest.org/index.php) strongly supports the key role of fish mitochondria in coping with different cellular stresses, such as hypoxia, low energy status and hypercortisolism [23]. However, the wide gene expression profiling of OXPHOS is far from being established in fish, and the first aim of the present study was to compile, revise and curate all the nucleotide sequences encoding for enzyme subunits of the mitochondrial respiratory chain in the recently updated gilthead sea bream transcriptomic database [24]. Secondly, we aimed to develop and validate a mitochondrial PCR-array for the comprehensive gene expression profiling of almost a complete set of assembly factors and enzyme complex subunits with either catalytic or regulatory properties on the basis of the available literature for orthologous genes in mammals and other fish species [20,25,26]. Thirdly, we sought to use this new genomic resource to achieve valuable insights into the tissue-specific regulation of OXPHOS by changing energy status upon fasting in liver and muscle tissues with either glycolytic (white skeletal muscle) or highly oxidative (heart) metabolic capabilities. At the protein level, the changes in gene expression were validated by Western blotting of cytochrome c oxidase subunit 4 (COX4). The final aim was to contribute to identifying the most responsive elements of the OXPHOS pathway for the tissue-specific phenotyping of nutritional and environmental metabolic disturbances of farmed fish and gilthead sea bream in particular.

## Material and Methods

### Sequence analysis

**Fig 1:**
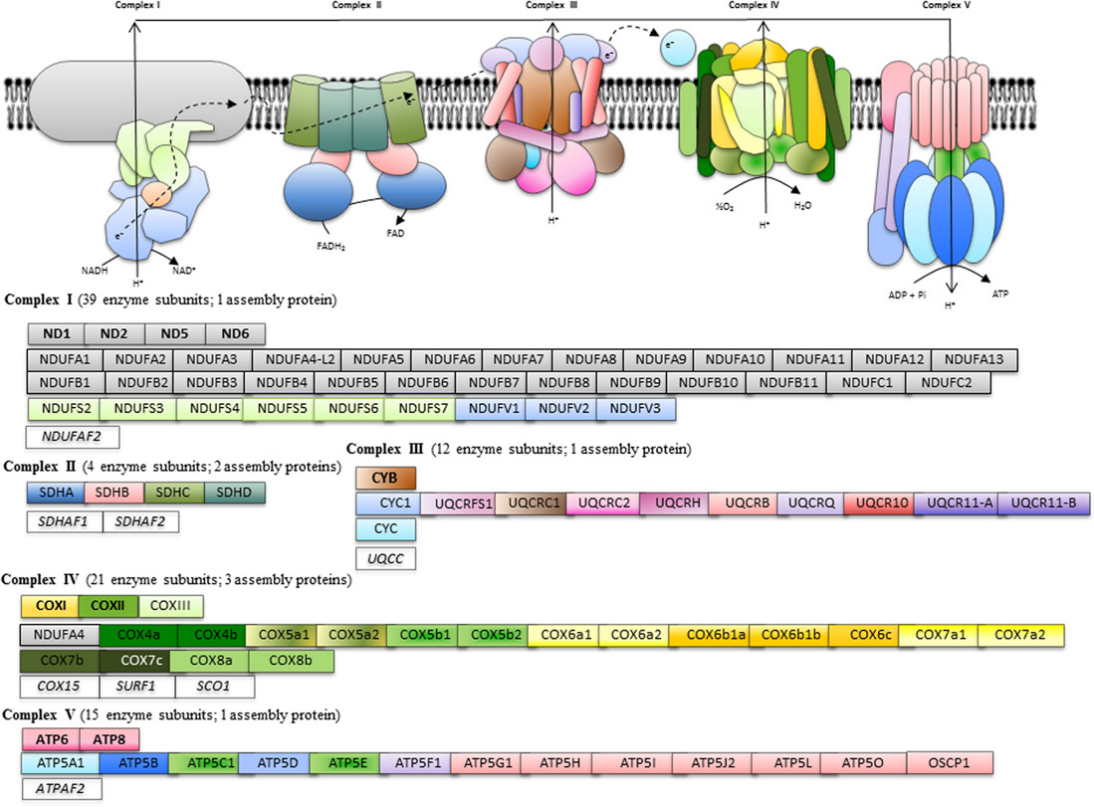
Schematic representation of annotated genes of the OXPHOS pathway in gilthead sea bream. Mitochondrial-encoded genes are highlighted in bold. Assembly factors are indicated in italics.

The gilthead sea bream transcriptomic database hosted at www.nutrigroup-iats.org/seabreamdb is highly enriched in mitochondrial-related genes with 926 non-redundant sequences with the Gene Ontology term “mitochondrion.” This allowed the unequivocal annotation of 99 sequences (E-values > 1e-15) as components of the KEGG pathway oxidative phosphorylation: 40 enzyme subunits and 1 assembly protein of Complex I, 4 enzyme subunits and 2 assembly proteins of Complex II, 12 enzyme subunits and 1 assembly protein of Complex III, 20 enzyme subunits and 3 assembly proteins of Complex IV, and 15 enzyme subunits and 1 assembly protein of Complex V as diagrammatically represented in Fig. 1. Ninety-seven out of 99 were new gilthead sea bream sequences with open reading frames of 159–1992 nucleotides in length and a variable number of reads (10–2349) composing the assembled sequences (S1–S5 Tables). All these sequences were uploaded to GenBank with accession numbers KC217558–KC217654. With the exception of the mitochondrial-encoded ATP synthase F0 subunit 6 (KC217599) and the nuclear-encoded protein OSCP1 (KC217613), all the uploaded sequences encode for complete coding regions.

### Fasting trial

Fish and samples to address the effect of fasting on the transcriptional regulation of OXPHOS come from a previous study [27]. Briefly, juvenile gilthead sea bream (*Sparus aurata* L.) of Atlantic origin (Ferme Marine de Douhet, Ile d’Oléron, France) were raised in the indoor experimental facilities of the Institute of Aquaculture Torre de la Sal (IATS). After an acclimation period of 3 months, fish with an average body weight of 86 g were distributed into 500 L tanks in 2 groups of 30 fish each. One group of fish continued to be fed with a commercial diet (EFICO YM 4.5, BioMar, Dueñas, Palencia, Spain) twice per day at full ration until visual satiety (CTRL group). The second group remained unfed for ten days. The feeding trial was conducted under natural photoperiod and temperature conditions at the latitude of the IATS (40°5N; 0°10E). Water flow was 20 L/min, the oxygen content of water effluents was always higher than 85% saturation, and unionized ammonia remained below toxic levels (<0.02 mg/L). At the end of the trial (following overnight fasting), eight randomly selected fish per dietary treatment were anesthetized with 3-aminobenzoic acid ethyl ester (MS-222, 100 μg/mL). Liver, white skeletal muscle (right-hand side) and heart ventricles were rapidly excised, frozen in liquid nitrogen and stored at −80°C until RNA extraction.

### Gene expression analysis

RNA from liver was extracted using a MagMAX-96 total RNA isolation kit (Life Technologies, Carlsbad, CA, USA). RNA yield was 50–100 μg with 260 and 280 nm UV absorbance ratios (A260/280) of 1.9–2.1, and RIN (RNA integrity number) values of 8–10 were measured on an Agilent 2100 Bioanalyzer, which is indicative of clean and intact RNA. Reverse transcription (RT) of 500 ng total RNA was performed with random decamers using a High-Capacity cDNA Archive Kit (Applied Biosystems, Foster City, CA, USA) according to the manufacturer’s instructions. Negative control reactions were run without reverse transcriptase and real-time quantitative PCR was carried out on an Eppendorf Mastercycler Ep Realplex Real-Time PCR Detection System (Eppendorf, Wesseling-Berzdorf, Germany).

The 96-well PCR-array layout was designed for the simultaneous profiling of a panel of 88 OXPHOS genes under uniform cycling conditions: 33 enzyme subunits and 1 assembly protein of Complex I, 4 enzyme subunits and 2 assembly proteins of Complex II, 12 enzyme subunits and 1 assembly protein of Complex III, 19 enzyme subunits and 3 assembly proteins of Complex IV, and 12 enzyme subunits and 1 assembly protein of Complex V (n = 12) (Table 1). Housekeeping genes and controls of general PCR performance were included in each array. All the pipetting and liquid manipulations required to perform the PCR-array were done by means of an EpMotion 5070 Liquid Handling Robot (Eppendorf, Hamburg, Germany) with no technical replicates in separate plates due to the very high data reproducibility. Briefly, RT reactions were diluted to convenient concentrations and the equivalent of 660 pg of total input RNA was used in a 25 μL volume for each PCR reaction. PCR wells contained a 2x SYBR Green Master Mix (Bio-Rad, Hercules, CA, USA), and specific primers at a final concentration of 0.9 μM were used to obtain amplicons of 50–150 bp in length (S6–S10 Tables). The program used for PCR amplification included an initial denaturation step at 95°C for 3 min, followed by 40 cycles of denaturation for 15 s at 95°C and annealing/extension for 60 s at 60°C. The efficiency of PCR reactions was always higher than 90% and similar for all the genes. Negative controls without sample templates were routinely performed for each primer set. The specificity of reactions was verified by analysis of melting curves (ramping rates of 0.5°C/10 s over a temperature range of 55–95°C), the linearity of serial dilutions of RT reactions, and electrophoresis and sequencing of PCR-amplified products.

**Table 1:** A. PCR-array layout (88 genes) with extra wells for housekeeping genes (ACTB) and general controls of PCR performance.

A												
	1	2	3	4	5	6	7	8	9	10	11	12
A	***ND2***	NDUFA6	NDUFB4	NDUFC2	**NDUFV3**	***CYB***	UQCRQ	COX4a	COX6b1b	*SCO1*	ATP5F1	PPC1
B	***ND5***	NDUFA7	NDUFB5	**NDUFS2**	*NDUFAF2*	CYCS	UQCR10	COX4b	COX6c1	*SURF1*	ATP5G1	PPC2
C	NDUFA1	NDUFA8	NDUFB6	**NDUFS4**	**SDHA**	**CYC1**	UQCR11-A	COX5a1	COX7a1	*COX15*	ATP5I	PPC3
D	NDUFA2	NDUFA9	NDUFB8	**NDUFS5**	**SDHB**	**UQCRFS1**	UQCR11-B	COX5a2	COX7a2	**ATP5A1**	ATP5J2	PPC4
E	NDUFA3	NDUFA12	NDUFB9	**NDUFS6**	SDHC	UQCRC1	*UQCC*	COX5b2	COX7b	**ATP5B**	ATP5L	NPC
F	NDUFA4	NDUFB1	NDUFB10	**NDUFS7**	SDHD	UQCRC2	***COXI***	COX6a1	COX7c	**ATP5C1**	ATP5O	ACTB
G	NDUFA4-like2	NDUFB2	NDUFB11	**NDUFV1**	*SDHAF1*	UQCRH	***COXII***	COX6a2	COX8a	**ATP5D**	OSCP	ACTB
H	NDUFA5	NDUFB3	NDUFC1	**NDUFV2**	*SDHAF2*	UQCRB	***COXIII***	COX6b1a	COX8b	**ATP5E**	*ATPAF2*	ACTB
**B**												
Position		Symbol		Description							Accession No.	
A1		***ND2***		NADH-ubiquinone oxidoreductase chain 2							KC217558	
B1		***ND5***		NADH-ubiquinone oxidoreductase chain 5							KC217559	
C1		NDUFA1		NADH dehydrogenase [ubiquinone] 1 alpha subcomplex subunit 1							KC217562	
D1		NDUFA2		NADH dehydrogenase [ubiquinone] 1 alpha subcomplex subunit 2							KC217563	
E1		NDUFA3		NADH dehydrogenase [ubiquinone] 1 alpha subcomplex subunit 3							KC217564	
F1		NDUFA4		NADH dehydrogenase [ubiquinone] 1 alpha subcomplex subunit 4							KC217565	
G1		NDUFA4-like2		NADH dehydrogenase [ubiquinone] 1 alpha subcomplex subunit 4-like 2							KC217566	
H1		NDUFA5		NADH dehydrogenase [ubiquinone] 1 alpha subcomplex subunit 5							KC217567	
A2		NDUFA6		NADH dehydrogenase [ubiquinone] 1 alpha subcomplex subunit 6							KC217568	
B2		NDUFA7		NADH dehydrogenase [ubiquinone] 1 alpha subcomplex subunit 7							KC217569	
C2		NDUFA8		NADH dehydrogenase [ubiquinone] 1 alpha subcomplex subunit 8							KC217570	
D2		NDUFA9		NADH dehydrogenase [ubiquinone] 1 alpha subcomplex subunit 9							KC217571	
E2		NDUFA12		NADH dehydrogenase [ubiquinone] 1 alpha subcomplex subunit 12							KC217574	
F2		NDUFB1		NADH dehydrogenase [ubiquinone] 1 beta subcomplex subunit 1							KC217576	
G2		NDUFB2		NADH dehydrogenase [ubiquinone] 1 beta subcomplex subunit 2							KC217577	
H2		NDUFB3		NADH dehydrogenase [ubiquinone] 1 beta subcomplex subunit 3							KC217578	
A3		NDUFB4		NADH dehydrogenase [ubiquinone] 1 beta subcomplex subunit 4							KC217579	
B3		NDUFB5		NADH dehydrogenase [ubiquinone] 1 beta subcomplex subunit 5							KC217580	
C3		NDUFB6		NADH dehydrogenase [ubiquinone] 1 beta subcomplex subunit 6							KC217581	
D3		NDUFB8		NADH dehydrogenase [ubiquinone] 1 beta subcomplex subunit 8							KC217583	
E3		NDUFB9		NADH dehydrogenase [ubiquinone] 1 beta subcomplex subunit 9							KC217584	
F3		NDUFB10		NADH dehydrogenase [ubiquinone] 1 beta subcomplex subunit 10							KC217585	
G3		NDUFB11		NADH dehydrogenase [ubiquinone] 1 beta subcomplex subunit 11							KC217586	
H3		NDUFC1		NADH dehydrogenase 1 subunit C1							KC217587	
A4		NDUFC2		NADH dehydrogenase 1 subunit C2							KC217588	
B4		**NDUFS2**		NADH dehydrogenase iron-sulfur protein 2							KC217589	
C4		**NDUFS4**		NADH dehydrogenase iron-sulfur protein 4							KC217591	
D4		**NDUFS5**		NADH dehydrogenase iron-sulfur protein 5							KC217592	
E4		**NDUFS6**		NADH dehydrogenase iron-sulfur protein 6							KC217593	
F4		**NDUFS7**		NADH dehydrogenase iron-sulfur protein 7							KC217594	
G4		**NDUFV1**		NADH dehydrogenase [ubiquinone] flavoprotein 1							KC217595	
A5		**NDUFV3**		NADH dehydrogenase [ubiquinone] flavoprotein 3							KC217597	
B5		*NDUFAF2*		NADH dehydrogenase (ubiquinone) 1 alpha subcomplex, assembly factor 2							KC217598	
C5		**SDHA**		Succinate dehydrogenase [ubiquinone] flavoprotein subunit							KC217615	
D5		**SDHB**		Succinate dehydrogenase [ubiquinone] iron-sulfur subunit							KC217616	
E5		SDHC		Succinate dehydrogenase cytochrome b560 subunit							KC217617	
F5		SDHD		Succinate dehydrogenase [ubiquinone] cytochrome b small subunit B							KC217618	
G5		*SDHAF1*		Succinate dehydrogenase assembly factor 1							KC217619	
H5		*SDHAF2*		Succinate dehydrogenase assembly factor 2							KC217620	
A6		***CYB***		Cytochrome b							DQ198005	
B6		CYCS		Cytochrome c							KC217632	
C6		**CYC1**		Cytochrome c1, heme protein							KC217621	
D6		**UQCRFS1**		Cytochrome b-c1 complex subunit Rieske							KC217622	
E6		UQCRC1		Cytochrome b-c1 complex subunit 1							KC217623	
F6		UQCRC2		Cytochrome b-c1 complex subunit 2							KC217624	
G6		UQCRH		Cytochrome b-c1 complex subunit 6							KC217625	
H6		UQCRB		Cytochrome b-c1 complex subunit 7							KC217626	
A7		UQCRQ		Cytochrome b-c1 complex subunit 8							KC217627	
B7		UQCR10		Cytochrome b-c1 complex subunit 9							KC217628	
C7		UQCR11-A		Cytochrome b-c1 complex subunit 10 isoform A							KC217629	
D7		UQCR11-B		Cytochrome b-c1 complex subunit 10 isoform B							KC217630	
E7		*UQCC*		Ubiquinol-cytochrome c reductase complex chaperone CBP3 homolog							KC217631	
F7		***COXI***		Cytochrome c oxidase subunit I							KC217652	
G7		***COXII***		Cytochrome c oxidase subunit II							KC217653	
H7		***COXIII***		Cytochrome c oxidase subunit II							KC217654	
A8		COX4a		Cytochrome c oxidase subunit 4 isoform 1							JQ308835	
B8		COX4b		Cytochrome c oxidase subunit 4 isoform 2							KC217633	
C8		COX5a1		Cytochrome c oxidase subunit 5A, mitochondrial-like isoform 1							KC217634	
D8		COX5a2		Cytochrome c oxidase subunit 5A, mitochondrial-like isoform 2							KC217635	
E8		COX5b2		Cytochrome c oxidase subunit 5B isoform 2							KC217637	
F8		COX6a1		Cytochrome c oxidase subunit 6A isoform 1							KC217638	
G8		COX6a2		Cytochrome c oxidase subunit 6A isoform 2							KC217639	
H8		COX6b1a		Cytochrome c oxidase subunit VIb isoform 1a							KC217640	
A9		COX6b1b		Cytochrome c oxidase subunit VIb isoform 1b							KC217641	
B9		COX6c1		Cytochrome c oxidase subunit 6C-1							KC217642	
C9		COX7a1		Cytochrome c oxidase subunit 7A1							KC217643	
D9		COX7a2		Cytochrome c oxidase subunit 7A2							KC217644	
E9		COX7b		Cytochrome c oxidase subunit 7B							KC217645	
F9		COX7c		Cytochrome c oxidase subunit 7C							KC217646	
G9		COX8a		Cytochrome c oxidase subunit 8A							KC217647	
H9		COX8b		Cytochrome c oxidase subunit 8B							KC217648	
A10		*SCO1*		SCO1 protein homolog, mitochondrial							KC217649	
B10		*SURF1*		Surfeit locus protein 1							KC217650	
C10		*COX15*		Cytochrome c oxidase assembly protein COX15 homolog							KC217651	
D10		**ATP5A1**		ATP synthase subunit alpha							KC217601	
E10		**ATP5B**		ATP synthase subunit beta							KC217602	
F10		**ATP5C1**		ATP synthase subunit gamma							KC217603	
G10		**ATP5D**		ATP synthase subunit delta							KC217604	
H10		**ATP5E**		ATP synthase subunit epsilon							KC217605	
A11		ATP5F1		ATP synthase subunit b							KC217606	
B11		ATP5G1		ATP synthase lipid-binding protein							KC217607	
C11		ATP5I		ATP synthase subunit e							KC217609	
D11		ATP5J2		ATP synthase subunit f							KC217610	
E11		ATP5L		ATP synthase subunit g							KC217611	
F11		ATP5O		ATP synthase subunit O							KC217612	
G11		OSCP		Protein OSCP1							KC217613	
H11		*ATPAF2*		Mitochondrial F1 complex assembly factor 2							KC217614	
A12-D12		PPC		Positive PCR control (serial dilutions of standard gene)							AY590304	
G12		NPC		Negative PCR control								
F12-H12		ACTB		ß-Actin							X89920	

**B.** Complete name and GenBank accession number for each gene in the OXPHOS array. Mitochondrial-encoded catalytic subunits are in bold and italics. Nuclear-encoded catalytic subunits are in bold. Nuclear-encoded regulatory subunits are in normal font. Nuclear-encoded assembly factors are in italics.

Fluorescence data acquired during the PCR extension phase were normalized using the delta-delta Ct method [28]. β-actin, elongation factor 1, α-tubulin and 18S rRNA were initially tested for gene expression stability using GeNorm software, but the most stable gene was β-actin (M score = 0.17) and, therefore, it was used as the housekeeping gene in the normalization procedure. When genes for a given nutritional condition were individually analyzed, fold-change calculations for each gene were in reference to the expression ratio between fasted and CTRL fish (values > 1 indicate fasting up-regulated genes; values < 1 indicate fasting down-regulated genes). For multi-gene analysis comparing mRNA gene expression level, all data values in a given tissue were in reference to the expression level in CTRL fish of NDUFC2 (liver), NDUFA5 (skeletal muscle) or NDUFB2 (heart), for which a value of 1 was arbitrarily assigned in the corresponding tissue.

### Western blotting

Samples for Western blotting were diluted with SDS-PAGE sample buffer (10% glycerol, 12.5% Tris base, 2% SDS, 0.05% bromophenol blue and 5% mercaptoethanol), boiled and centrifuged at 13,000 g for 10 min. The supernatants were decanted and equal amounts of protein (20 μg) were layered and electroblotted as reported elsewhere [29]. Briefly, blots were incubated with a polyclonal rabbit antiserum raised against human COX4 (ab16056, Abcam, Cambridge, UK) diluted at 1:2000. This antibody is directed to a 19 amino acid epitope (NPIQGLASKWDYEKNEWKK) from within residues 150 to the C-terminus of human COX4, sharing a homology of 78% and 88% with gilthead sea bream COX4a and COX4b, respectively. Detection of signal was done using an enhanced chemiluminiscence system (Santa Cruz Biotechnology, Santa Cruz, CA, USA) and a VersaDoc Model 5000 imaging system (Bio-Rad). Prestained markers (Fermentas, Burlington, Canada) were used to estimate the size and position of protein in the gel.

### Statistical analyses

Fasting-mediated effects on growth performance and tissue mRNA transcripts were analyzed by Student t-test at a significance level of 5%. All analyses were made using the SPSS package version 20.0 (SPSS Inc., Chicago, IL, USA).

### Ethics statement

All procedures were approved by the Ethics and Animal Welfare Committee of Institute of Aquaculture Torre de la Sal and carried out in a registered installation (code 36271-42-A) in accordance with the principles published in the European animal directive (2010/63/EU) and Spanish laws (Royal Decree RD53/2013) for the protection of animals used in scientific experiments. In all lethal samplings, fish were decapitated under 3-aminobenzoic acid ethyl ester (MS-222, 100 μg/mL) anesthesia, and all efforts were made to minimize suffering.

## Results

### Fish performance

As shown in Table 2, continuously fed fish (CTRL) grew efficiently with an 18–20% increase in body weight, while fasted fish lost 6–8% of body weight mass over the course of the 10-day fasting period. The viscera weight and liver weight of fasted fish were significantly lower than those of CTRL fish, and the resulting viscerosomatic and hepatosomatic indexes decreased from 8.5% to 5.4% and from 2.1% to 0.6%, respectively.

**Table 2:** Growth and biometric parameters of fed (CTRL group) and fasted gilthead sea bream.

	CTRL	Fasted	*P* ^a^
Final body weight (g)	109.48 ± 3.42	79.93 ± 1.82	*<0*.*001*
Viscera (g)	9.35 ± 0.49	4.34 ± 0.23	*<0*.*001*
Liver (g)	2.31 ± 0.13	0.52 ± 0.03	*<0*.*001*
VSI (%) ^b^	8.52 ± 0.23	5.41 ± 0.19	*<0*.*001*
HSI (%) ^c^	2.10 ± 0.06	0.64 ± 0.02	*<0*.*001*
DM intake (g/fish)	17.25	-	

Each value is the mean ± SEM of the 8 sampled fish for transcriptional analysis. Initial average weight for the entire population was 86 ± 0.08 g.

^a^P values result from Student-t test.

^b^Viscerosomatix index = (100 × viscera wt.) / fish wt.

^c^Hepatosomatic index = (100 × liver wt.) / fish wt.

### Gene expression profiling

Complete data on liver, white skeletal muscle and heart gene expression are shown in S11 Table. As a general rule, fasting produced a down-regulated response of OXPHOS in the liver tissue, which was statistically significant for 80% of the genes present in the array (72 out of 88). In contrast, a statistically significant up-regulated response was found for 29 and 10 genes in white skeletal muscle and the heart, respectively. Overall, in each tissue the magnitude of change paralleled the number of differentially expressed genes, and the multiplier factor for the average fold-change of differentially expressed genes was 0.5 in the liver, 1.7 in white skeletal muscle and 1.5 in the heart.

**Fig 2:**
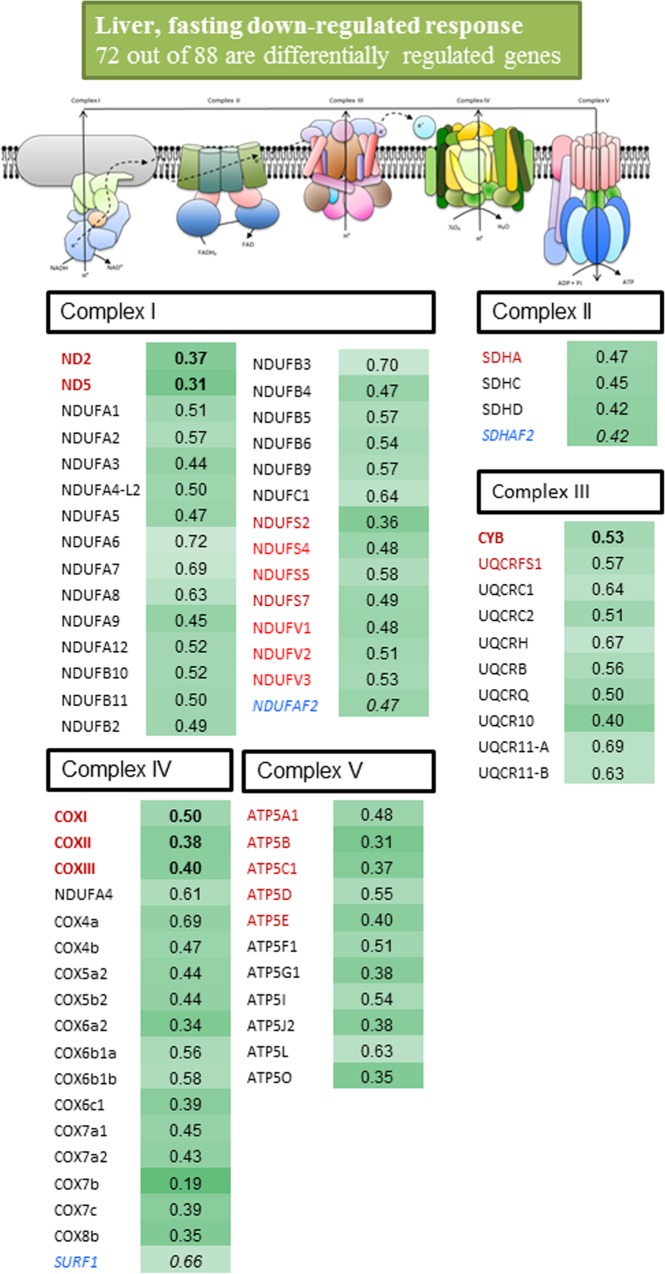
Fold-change of differentially expressed genes (P< 0.05) in the liver tissue of fasted fish. Fish were fed with a commercial diet to visual satiety (Control, CTRL group) or remained unfed for ten days (fasted group). Data of fold-change are relative to the CTRL group. The intensity of green boxes represents the degree of down-regulation. Mitochondrial-encoded catalytic subunits are in bold and red. Nuclear-encoded catalytic subunits are in red. Nuclear-encoded regulatory subunits are in black. Nuclear-encoded assembly factors are in blue and italics.

**Fig 3:**
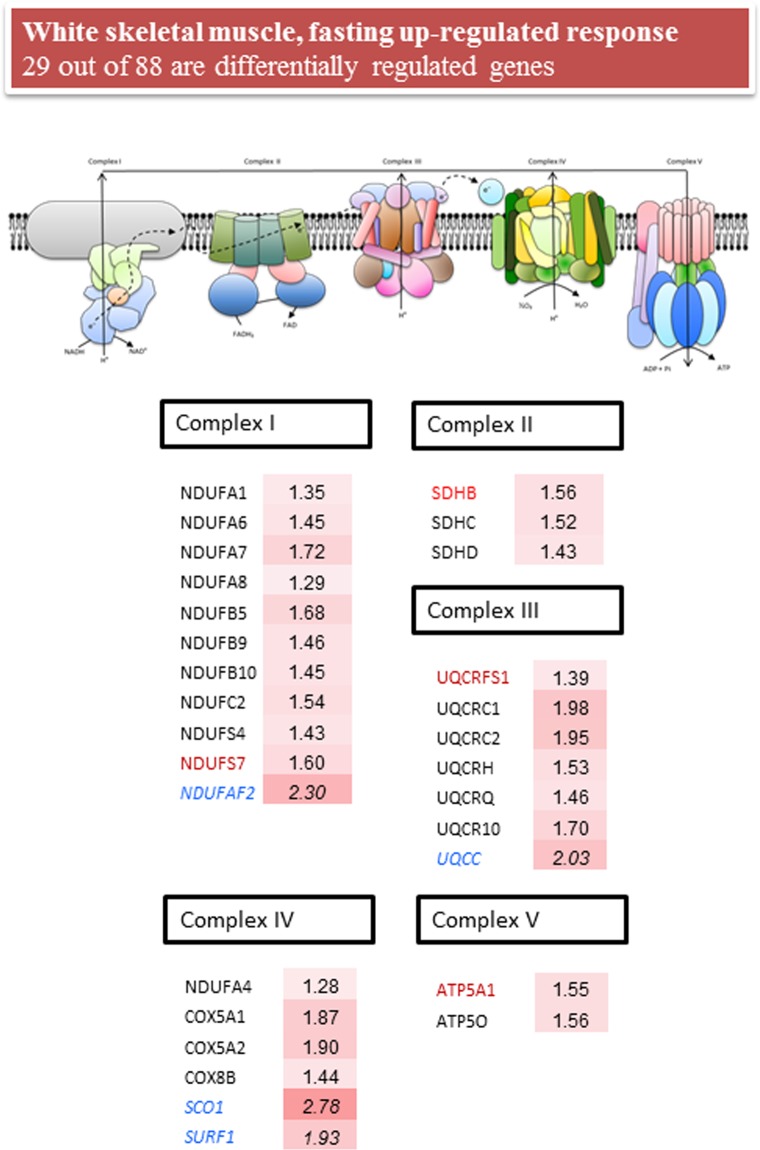
Fold-change of differentially expressed genes (P< 0.05) in the white skeletal muscle of fasted fish. Fish were fed with a commercial diet to visual satiety (Control, CTRL group) or remained unfed for ten days (fasted group). Data of fold-change are relative to the CTRL group. The intensity of red boxes represents the degree of up-regulation. Nuclear-encoded catalytic subunits are in red. Nuclear-encoded regulatory subunits are in black. Nuclear-encoded assembly factors are in blue and italics.

**Fig 4:**
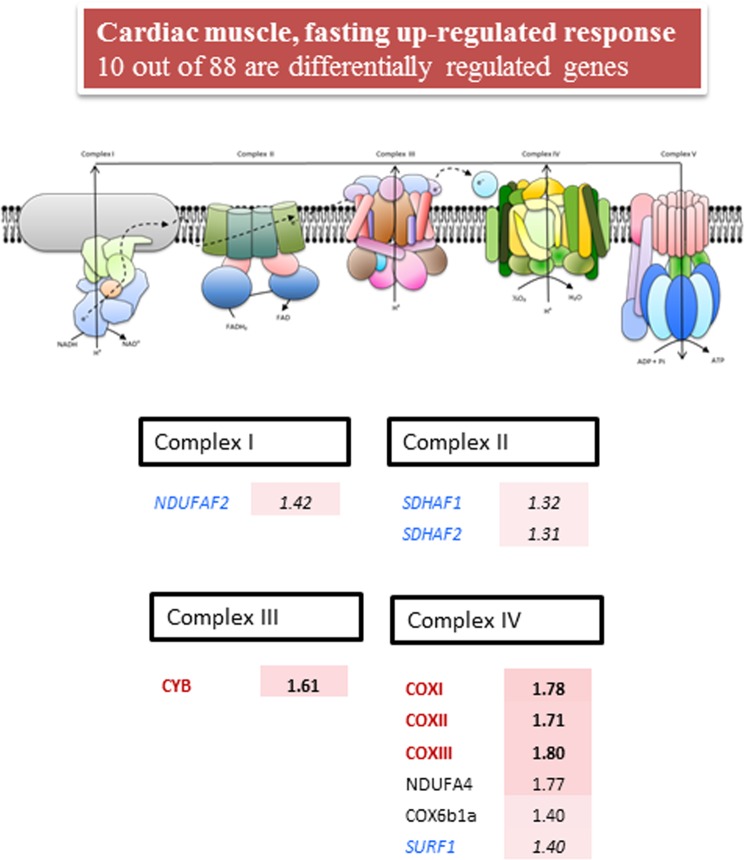
Fold-change of differentially expressed genes (P< 0.05) in the cardiac muscle of fasted fish. Fish were fed with a commercial diet to visual satiety (Control, CTRL group) or remained unfed for ten days (fasted group). Data of fold-change are relative to the CTRL group. The intensity of red boxes represents the degree of up-regulation. Mitochondrial-encoded catalytic subunits are in bold and red. Nuclear-encoded catalytic subunits are in red. Nuclear-encoded regulatory subunits are in black. Nuclear-encoded assembly factors are in blue and italics.

For a better understanding of expression data, differentially expressed genes with a fold-change cutoff of 1.25 and 0.8 were compiled and graphically represented for each tissue in Figs. 2–4. In the liver (Fig. 2), the magnitude of change was of the same order within and among all the components of the respiratory chain, encoded either by mtDNA or nDNA. Thus, 29 out of 33 sequences of Complex I, including catalytic (ND2, ND5, NDUFS2, NDUFS4, NDUFS5, NDUFS7, NDUFV1-3), regulatory (NDUFA1-3, NDUFA5-9, NDUFA12, NDUFB2-6, NDUFB9-11, NDUFC1) and assembly factors (NDUFAF2), were significantly down-regulated with fold-changes of 0.3–0.7. Complex II was also consistently and significantly down-regulated (4 out of 6 sequences) with fold-changes of 0.4–0.5 for catalytic (SDHA), regulatory (SDHC, SDHD) and assembly factors (SDHAF2). Two catalytic (CYB, UQCRFS1) and 8 regulatory (UQCRC1-2, UQCRH, UQCRB, UQCRQ, UQCR10, UQCR11-A, UQCR11-B) subunits of Complex III (10 out of 13) were significantly down-regulated with fold-changes of 0.4–0.7. Complex IV was also extensively down-regulated (18 out of 23 subunits) with fold-changes varying between 0.2 and 0.7 for catalytic (COXI-III), regulatory (NDUFA4, COX4a,-b, COX5a2, COX5b2, COX6a2, COX6b1a-b, COX6c1, COX7a1-2, COX7b-c, COX8b) and assembly (SURF1) factors. Finally, 5 catalytic (ATP5A1, ATP5B, ATP5C1, ATP5D, ATP5E) and 6 regulatory (ATP5F1, ATP5G1, ATP5I, ATP5J2, ATP5L, ATP5O) elements of Complex V (11 out of 13) were significantly down-regulated by nutrient intervention with fold-changes of 0.3–0.6.

As shown in Fig. 3, 11 nuclear-encoded subunits of Complex I with catalytic (NDUFS4, NDUFS7), regulatory (NDUFA1, NDUFA6-7, NDUFB5, NDUFB9-10, NDUFC2) and assembly (NDUFAF2) functions were consistently up-regulated (fold-change 1.3–2.3) by fasting in white skeletal muscle, but we failed to detect consistent changes in catalytic mitochondrial-encoded elements. Complex II was entirely encoded by nDNA and a consistent up-regulation was found for catalytic (SDHB) and regulatory (SDHC, SDHD) subunits with fold changes of 1.4–1.6. It was the same for Complex III and IV with a significant up-regulation of 13 nuclear transcripts encoding for catalytic (UQCRFS1), regulatory (UQCRC1-2, UQCRH, UQCRQ, UQRC10, NDUFA4, COX5A1-2, COX8B) and assembly factors (UQCC, SCO1, SURF1) with fold-changes varying between 1.3 and 2.8, but again no consistent changes were found for the mitochondrial-encoded subunits. Less evident were the transcriptionally mediated effects on Complex V, and a consistent up-regulation with fold changes of 1.5 was only found for the nuclear-encoded ATP5A1 and ATP5O.

In the heart (Fig. 4), the number of differentially regulated genes of OXPHOS was drastically reduced to 10 with overrepresentation of nuclear-encoded assembly factors (Complex I, NDUFAF2; Complex II, SDHAF1-2; Complex IV, SURF1) and mitochondrial-encoded elements (Complex III, CYB; Complex IV, COXI, COXII, COXIII) with fold-changes of 1.3–1.4 and 1.6–1.8, respectively.

### COX4 protein levels

**Fig 5:**
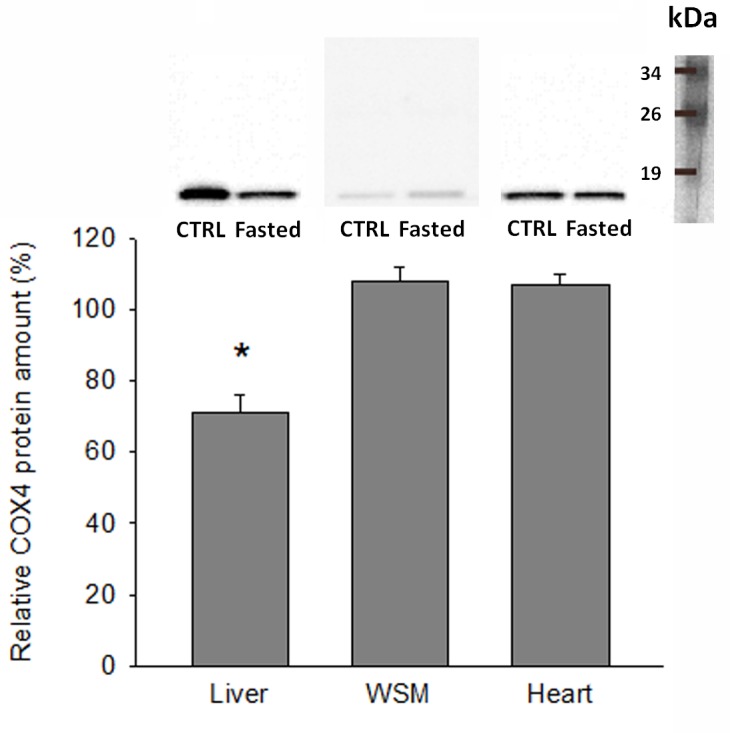
Western blot of COX4 in liver, white skeletal muscle and cardiac muscle of CTRL and fasted fish. Representative Western blots of tissue protein samples (20 μg) of CTRL and fasted individuals, and integrated intensities of bands. For each tissue, data are expressed as the percentage of intensity in comparison with the CTRL group samples (100% value). Data are represented as mean ± SEM (n = 6) and statistically significant differences between CTRL and fasted groups are indicated (*, P<0.05; Student t-test).

Western blot of tissue extracts with the COX4 antibody revealed a protein band of expected size (approximately 20 kDa) in both liver and muscle tissue samples. Of note, COX4 protein levels were significantly decreased by fasting in the liver tissue (70% CTRL values), paralleling the changes observed by mRNA gene expression analysis. In contrast, a slight increase was found in heart and white skeletal muscle, although both at the protein and mRNA levels the fasting-induced changes were not statistically significant (Fig. 5).

## Discussion

Mitochondrial OXPHOS provides over 90% of the ATP produced by mammalian cells, and, therefore, the number of mitochondria and their level of activity vary with the tissue and cell type reflecting the energy requirements of the cell [30,31]. This also applies to fish, and the expression profile of selected markers of mitochondrial dynamics and apoptosis, mitochondrial protein import, folding and assembly, and mitochondrial biogenesis and oxidative metabolism mirror the intensity and severity of natural and husbandry stressors in farmed gilthead sea bream [22]. Previous studies on gilthead sea bream also indicate that the mitochondrial “allostatic load” is altered by dietary oils in crowded stressed fish [21], and overall we consider that stressful and health risk factors segregate with the low expression levels of genes required for mitochondrial biogenesis and OXPHOS as previously reported in higher vertebrates [32]. Furthermore, experimental evidence in gilthead sea bream [33,34] and other fish species [35,36] indicates that hypoxia and nutrient (metabolic fuel) overflow activate the futile cycle of energy production via the increased expression of uncoupling respiratory proteins (UCP1–3) to match the antioxidant defense system. However, as pointed out before, the fine regulation of OXPHOS is not yet established, and the present study provides new and valuable insights into how gilthead sea bream mitochondria are modulated in a tissue-specific manner to cope with the altered metabolic needs upon starvation. This includes the uploading to public repository databases of almost a complete set of OXPHOS genes (97 new gilthead sea bream sequences), which allowed a new and powerful genomic resource to be developed for a comprehensive transcriptomic profiling of the mitochondrial respiratory chain in a marine farmed fish species of a high added value.

Complex I is the largest among the mitochondrial respiratory chain and varies from 14 subunits in prokaryotes to 45 subunits in mammals [37–39]. In the present study, we unequivocally annotated up to 40 new enzyme subunits, including among them four mtDNA-encoded subunits (ND1, ND2, ND5, ND6), six iron-sulphur proteins (NDUFS2-7), three flavoprotein subunits (NDUFV1-3), 13 regulatory subunits of the alpha subcomplex (NDUFA1-3, NDUFA5-13, NDUFA4-L2; NDUFA4 has been considered as a subunit of complex IV as recently reported by [40]), 11 regulatory subunits of the beta subcomplex (NDUFB1-11) and the two subunits of the NDUFC complex (NDUFC1 and NDUFC2), in addition to the essential assembly factor NDUFAF2/mimitin [41]. Two assembly factors (SDHAF1-2) and four nDNA-encoded enzyme subunits of Complex II with either catalytic (SDHA-B) or regulatory (SDHC-D) properties were also recognized and properly annotated [42]. Likewise, Complex III is composed of 12 enzyme subunits and all of them, with the exception of cytochrome b (CYB), are encoded by nDNA [43]. Importantly, all these enzyme subunits are conserved in gilthead sea bream, and together with two enzyme isoforms of the regulatory subunit UQCR11 (UQCR11-A, UQCR11-B) they have been identified as actively transcribed genes in a typical marine fish.

Complex IV is composed of a variable number of enzyme subunits (4–13) [44,45], and the catalytic core represented by the mtDNA-encoded COXI, COXII and COXIII is already found in our transcriptomic gilthead sea bream database. This enzyme complex is the most studied, and early studies in sheep, dogs, rabbits, rats, mice and humans share a characteristic gene expression pattern on the basis of the species [46], tissue [47] and developmental stage [48]. In the present study, up to 20 enzyme subunits of Complex IV were annotated, including 16 conserved vertebrate paralogs of COX4 (COX4a, COX4b), COX5a (COX5a1, COX5a2), COX5b (COX5b1, COX5b2), COX6a (COX6a1, COX6a2), COX6b (COX6b1, COX6b2), COX6c (COX6c1, COX6c2), COX7a (COX7a1, COX7a2), COX8 (COX8a, COX8b) and five fish species-specific subunits annotated as COX6b1a, COX6b1b, COX6c1, COX7b and COX7c [49]. Additionally, we annotated for the first time in a non-model fish species the assembly factors COX15, SCO1 and SURF1, which are essential for the normal function of the enzyme complex [50]. Indeed, COX15 converts heme O into heme A by hydroxylation, which is then incorporated during early assembly into Complex IV, and any mutation in COX15 leads to the arrest and degradation of the complex [51]. Likewise, SCO1 is involved in cellular copper homeostasis, and mutations in SCO1 cause a neonatal hepatopathy and ketoacidotic coma [52]. In humans and flies, mutations in SURF1 are generally lethal, but paradoxically SURF1 knockouts are associated with prolonged longevity and neuroprotection in mice [53].

Complex V comprises a catalytic sector (F_1_), a membrane sector (F_0_) and a long stalk connecting F_1_ to F_0_. Out of a total of 15 subunits, two (ATP6 and ATP8) are encoded by mtDNA and the remaining by nDNA [54,55], and all of them, including the F_1_-stator (ATP5A1, B, C), the rotor (ATP5D, E, ATP5G) and the proton translocation of the F_0_ sector, comprising the membrane stator (ATP6, ATP8), the stator-peripheral stalk (ATP5F1, ATP5H, ATP5J2, ATPO, OSCP) and the dimerization subunits (ATP5I, ATP5L), were properly annotated. The ATPAF2 assembly factor, required for the correct function of Complex V [26], was also identified, which confirms and extends the notion that catalytic, regulatory and assembly factors of OXPHOS have been highly conserved through the evolution of fish and higher vertebrate species with a differential and tissue-specific regulation in fish exposed to different metabolic stressors as reported below.

From a functional point of view, it is noteworthy that in our fasting model most of the components of our OXPHOS array were significantly down-regulated in the liver tissue. The magnitude of change was of the same order of magnitude for all the enzyme complexes (Complex I–V), and importantly this massive response included catalytic enzyme subunits, encoded either by mtDNA (ND2, ND5, CYB, COXI-III) or nDNA (NDUFS2, NDUFS4, NDUFS5, NDUFS7, NDUFV1-3, SDHA, UQCRFS1, ATP5A1, ATP5B, ATP5C1, ATP5D, ATP5E), and nuclear-encoded regulatory enzyme subunits (NDUFA1-9, NDUFA12, NDUFB2-6, NDUFB9-11, NDUFC1, SDHC, SDHD, UQCRC1-2, UQCRH, UQCRB, UQCRQ, UQCR10, UQCR11-B, COX4a,-b, COX5a2, COX5b2, COX6a2, COX6b1a-b, COX6c1, COX7a1-2, COX7b-c, COX8b, ATP5F1, ATP5G1, ATP5I, ATP5J2, ATP5L, ATP5O) and nuclear-encoded assembly factors (NDUFAF2, SDHAF2, SURF1) as well. This consistent response substantiates a reduced energy demand as the result of the fasting inhibition of hepatic lipogenesis, which is considered a major energy-demanding process in the liver tissue [56]. Hence, we found herein a marked loss of adipose tissue mass and liver size, which is concurrent with a strong down-regulation of a vast array of hepatic lipogenic enzymes, including fatty acid elongases (ELOVL4, ELOVL5, ELOVL6) and desaturases with Δ6 (FASD2) and Δ9 (SCD1a and SCD1b) activities [27]. In the present study, additional evidence for all this is supported by the observation that the expression of COX4 subunit isoforms was dampened by fasting at both the mRNA and protein level. Fasting or caloric restriction also down-regulate OXPHOS and the TCA cycle in the liver tissue of pigs [57], mice [58] and chickens [59]. A similar trend was reported for the liver of European eels after exposure to environmental pollutants [16,17], although reliable results were reduced to regulatory enzyme subunits due to the poor representation of assembly factors and catalytic enzyme subunits of OXPHOS in the arrays used for the gene expression profiling

In fish, switches in muscle energy demand or oxidative capacities are often related to intensity training [60] or long fasting spawning migrations [18,61]. However, nutrient availability by itself is a major factor driving switches in muscle protein turnover and mitochondrial activity as reported earlier in gilthead sea bream [23] by microarray gene expression profiling of glycolytic and aerobic muscle tissues in fish fed to maintenance ration. This is consistent with the up-regulation of OXPHOS in white skeletal muscle and the heart, although both in this and previous studies in pigs [62] and mice [63] the response of skeletal and cardiac muscle tissues to food deprivation and/or restriction is not only opposite to, but also weaker than, in the liver. This notion was substantiated herein by the magnitude of fold-change and the number of differentially expressed genes, which was reduced from 72 in the liver to 29 and 10 in skeletal muscle and cardiac muscle, respectively. Furthermore, it should be noted that the response of skeletal muscle was mostly mediated by regulatory and assembly factors encoded by mitochondrial DNA, whereas that of cardiac muscle was mostly due to catalytic and assembly factors encoded by mitochondrial and nuclear DNA. In humans, a differential response of mitochondrial complexes has also been found with age in skeletal muscle, with a decrease in gene transcripts for several components of complexes I, IV and V, and no major changes for complexes II and III [64,65]. The physiological significance of these findings is far from being fully established, although they can be viewed as a different tissue-metabolic plasticity of glycolytic and highly oxidative muscle tissues, which was encompassed in a complex manner by the nuclear and mitochondrial genomes. As reported for liver, changes in mRNA gene expression fit well with the Western blotting of COX4, although further research is needed to assess with commercial and customized antibodies the concurrent protein changes of the most transcriptionally regulated OXPHOS subunits in front of a wide range of physiological challenges.

## Conclusions

The molecular identity of almost all the components of the mitochondrial respiratory chain has been established for the first time in a non-model fish species. This yielded 97 new gilthead sea bream sequences, all of them manually curated and uploaded to GeneBank. This allowed the development of a powerful PCR-array, which has been used with success for the simultaneous expression profiling of 88 OXPHOS genes with catalytic, regulatory and assembly properties. Most of them are becoming highly regulated genes by nutrient deprivation in the liver tissue, whereas a moderate or low response was found for the glycolytic skeletal muscle and the highly oxidative cardiac muscle, respectively. The direction of change is also tissue-specific, according to the different metabolic capabilities of liver and muscle tissues. These findings contribute to refining the list of candidate genes for phenotyping any metabolic disturbance in farmed fish and gilthead sea bream in particular. Whether this is fish species-specific remains to be resolved, although we suspect that it is part of the highly conserved metabolic features through the evolution of fish and high vertebrate species, which is prone to conserve the complex interactions of mitochondrial and nuclear genomes.

## Supporting Information

S1 Table Characteristics of the new gilthead sea bream assembled sequences of Complex I. (DOCX)

S2 Table Characteristics of the new gilthead sea bream assembled sequences of Complex II. (DOCX)

S3 Table Characteristics of the new gilthead sea bream assembled sequences of Complex III. (DOCX)

S4 Table Characteristics of the new gilthead sea bream assembled sequences of Complex IV. (DOCX)

S5 Table Characteristics of the new gilthead sea bream assembled sequences of Complex V. (DOCX)

S6 Table Forward and reverse primers for real-time PCR of Complex I. (DOCX)

S7 Table Forward and reverse primers for real-time PCR of Complex II. (DOC)

S8 Table Forward and reverse primers for real-time PCR of Complex III. (DOCX)

S9 Table Forward and reverse primers for real-time PCR of Complex IV. (DOCX)

S10 Table Forward and reverse primers for real-time PCR of Complex V. (DOCX)

S11 Table Relative gene expression of OXPHOS genes in liver, white skeletal muscle (WSM) and heart of control and fasted fish. (DOCX)
