# Supplementary material for: Unraveling the Molecular Signatures of Oxidative Phosphorylation to Cope with the Nutritionally Changing Metabolic Capabilities of Liver and Muscle Tissues in Farmed Fish
Source: PLoS One. 2015 Apr 15;10(4):e0122889. doi: 10.1371/journal.pone.0122889 (PMC4398389; doi:10.1371/journal.pone.0122889)
Supplement: S1 Table — (DOCX) [file pone.0122889.s001.docx]

**Supporting information Table S1**. **Characteristics of the new gilthead sea bream assembled sequences of Complex I**. Mitochondrial-encoded catalytic subunits are in bold and red. Nuclear-encoded catalytic subunits are in red. Nuclear-encoded regulatory subunits are in black. Nuclear-encoded assembly factors are in blue and italics.

| Contigs | F^a^ | Size (nt) | Annotation^b^ | Best match^c^ | E^d^ | CDS^e^ | Accession No^f^ |
| --- | --- | --- | --- | --- | --- | --- | --- |
| C2_1453 | 555 | 1036 | **ND1** | YP_001256939 | 0 | 9-983 | KC217558 |
| C2_994 | 706 | 1086 | **ND2** | YP_001974735 | 5e-135 | 7-1062 | KC217559 |
| C2_87 | 2349 | 2499 | **ND5** | YP_001256949 | 0 | 73-1914 | KC217560 |
| C2_2144 | 142 | 812 | **ND6** | YP_001256950 | 2e-80 | 73-594 | KC217561 |
| C2_8082 | 80 | 587 | NDUFA1 | ACQ58283 | 6e-38 | 123-335 | KC217562 |
| C2_1985 | 179 | 468 | NDUFA2 | CAF92313 | 6e-55 | 46-348 | KC217563 |
| C2_4897 | 71 | 431 | NDUFA3 | XP_00340957 | 1e-43 | 72-314 | KC217564 |
| C2_1577 | 173 | 1230 | NDUFA4-like2 | XP_003444820 | 2e-46 | 120-368 | KC217566 |
| C2_22864 | 63 | 590 | NDUFA5 | XP_003455688 | 2e-64 | 47-397 | KC217567 |
| C2_9313 | 102 | 607 | NDUFA6 | CAG01654 | 2e-64 | 47-433 | KC217568 |
| C2_2419 | 111 | 580 | NDUFA7 | XP_003449062 | 2e-61 | 52-378 | KC217569 |
| C2_1373 | 277 | 1027 | NDUFA8 | XP_003444379 | 2e-116 | 161-679 | KC217570 |
| C2_332 | 905 | 1301 | NDUFA9 | ACQ58220 | 0 | 48-1193 | KC217571 |
| C2_1225 | 484 | 1334 | NDUFA10 | XP_003457387 | 0 | 87-1154 | KC217572 |
| C2_4493 | 81 | 523 | NDUFA11 | XP_003448809 | 1e-83 | 41-448 | KC217573 |
| C2_110117 | 42 | 549 | NDUFA12 | XP_003448116 | 2e-68 | 27-470 | KC217574 |
| C2_121074 | 116 | 596 | NDUFA13 | XP_003438233 | 2e-93 | 50-484 | KC217575 |
| C2_9246 | 57 | 401 | NDUFB1 | CAG03553 | 1e-31 | 73-249 | KC217576 |
| C2_15870 | 122 | 553 | NDUFB2 | ACI68020 | 7e-58 | 126-434 | KC217577 |
| C2_15466 | 38 | 448 | NDUFB3 | XP_003457472 | 2e-55 | 81-362 | KC217578 |
| C2_3428 | 100 | 505 | NDUFB4 | XP_003443537 | 3e-50 | 38-421 | KC217579 |
| C2_467 | 696 | 702 | NDUFB5 | ACQ58904 | 7e-97 | 45-602 | KC217580 |
| C2_3928 | 73 | 588 | NDUFB6 | XP_003452285 | 1e-80 | 106-489 | KC217581 |
| C2_1170 | 317 | 614 | NDUFB7 | XP_003450021 | 6e-65 | 162-530 | KC217582 |
| C2_1305 | 310 | 685 | NDUFB8 | XP_003441843 | 5e-89 | 48-623 | KC217583 |
| C2_3902 | 244 | 958 | NDUFB9 | XP_003458631 | 6e-117 | 55-582 | KC217584 |
| C2_497 | 427 | 925 | NDUFB10 | XP_003456422 | 3e-105 | 236-751 | KC217585 |
| C2_6703 | 101 | 572 | NDUFB11 | XP_003454298 | 4e-77 | 61-498 | KC217586 |
| C2_5740 | 92 | 518 | NDUFC1 | ACQ58409 | 4e-30 | 191-400 | KC217587 |
| C2_4941 | 159 | 617 | NDUFC2 | ACO09452 | 8e-49 | 143-475 | KC217588 |
| C2_961 | 352 | 1581 | NDUFS2 | ACQ58471 | 0 | 26-1426 | KC217589 |
| C2_1740 | 354 | 919 | NDUFS3 | XP_003456935 | 6e-161 | 37-819 | KC217590 |
| C2_1717 | 308 | 648 | NDUFS4 | XP_003451066 | 3e-96 | 46-555 | KC217591 |
| C2_9639 | 39 | 515 | NDUFS5 | XP_003454647 | 1e-48 | 83-403 | KC217592 |
| C2_10276 | 92 | 486 | NDUFS6 | XP_003443820 | 2e-76 | 23-412 | KC217593 |
| C2_1860 | 262 | 1160 | NDUFS7 | XP_003449069 | 1e-133 | 46-705 | KC217594 |
| C2_675 | 598 | 1690 | NDUFV1 | XP_003452502 | 0 | 95-1507 | KC217595 |
| C2_3103 | 204 | 905 | NDUFV2 | ACQ58245 | 0 | 84-818 | KC217596 |
| C2_4224 | 86 | 552 | NDUFV3 | XP_003456249 | 9e-15 | 38-361 | KC217597 |
| C2_1316 | 682 | 1007 | *NDUFAF2* | XP_003446117 | 2e-88 | 119-607 | KC217598 |

^a^Number of reads composing the assembled sequences.

^b^Gene identity determined through BLAST searches: ND1, NADH-ubiquinone oxidoreductase chain 1; ND2, NADH-ubiquinone oxidoreductase chain 2; ND5, NADH-ubiquinone oxidoreductase chain 5; ND6, NADH dehydrogenase subunit 6. NDUFA1, NADH dehydrogenase [ubiquinone] 1 alpha subcomplex subunit 1; NDUFA2, NADH dehydrogenase [ubiquinone] 1 alpha subcomplex subunit 2; NDUFA3, NADH dehydrogenase [ubiquinone] 1 alpha subcomplex subunit 3; NDUFA4-like2, NADH dehydrogenase [ubiquinone] 1 alpha subcomplex subunit 4-like 2; NDUFA5, NADH dehydrogenase [ubiquinone] 1 alpha subcomplex subunit 5; NDUFA6, NADH dehydrogenase [ubiquinone] 1 alpha subcomplex subunit 6; NDUFA7, NADH dehydrogenase [ubiquinone] 1 alpha subcomplex subunit 7; NDUFA8, NADH dehydrogenase [ubiquinone] 1 alpha subcomplex subunit 8; NDUFA9, NADH dehydrogenase [ubiquinone] 1 alpha subcomplex subunit 9; NDUFA10, NADH dehydrogenase [ubiquinone] 1 alpha subcomplex subunit 10; NDUFA11, NADH dehydrogenase [ubiquinone] 1 alpha subcomplex subunit 11; NDUFA12, NADH dehydrogenase [ubiquinone] 1 alpha subcomplex subunit 12; NDUFA13, NADH dehydrogenase [ubiquinone] 1 alpha subcomplex subunit 13; NDUFB1, NADH dehydrogenase [ubiquinone] 1 beta subcomplex subunit 1; NDUFB2, NADH dehydrogenase [ubiquinone] 1 beta subcomplex subunit 2; NDUFB3, NADH dehydrogenase [ubiquinone] 1 beta subcomplex subunit 3; NDUFB4, NADH dehydrogenase [ubiquinone] 1 beta subcomplex subunit 4; NDUFB5, NADH dehydrogenase [ubiquinone] 1 beta subcomplex subunit 5; NDUFB6, NADH dehydrogenase [ubiquinone] 1 beta subcomplex subunit 6; NDUFB7, NADH dehydrogenase [ubiquinone] 1 beta subcomplex subunit 7; NDUFB8, NADH dehydrogenase [ubiquinone] 1 beta subcomplex subunit 8; NDUFB9, NADH dehydrogenase [ubiquinone] 1 beta subcomplex subunit 9; NDUFB10, NADH dehydrogenase [ubiquinone] 1 beta subcomplex subunit 10; NDUFB11, NADH dehydrogenase [ubiquinone] 1 beta subcomplex subunit 11; NDUFC1, NADH dehydrogenase 1 subunit C1; NDUFC2, NADH dehydrogenase 1 subunit C2; NDUFS2, NADH dehydrogenase iron-sulfur protein 2; NDUFS3, NADH dehydrogenase iron-sulfur protein 3; NDUFS4, NADH dehydrogenase iron-sulfur protein 4; NDUFS5, NADH dehydrogenase iron-sulfur protein 5; NDUFS6, NADH dehydrogenase iron-sulfur protein 6; NDUFS7, NADH dehydrogenase iron-sulfur protein 7; NDUFV1, NADH dehydrogenase [ubiquinone] flavoprotein 1; NDUFV2, NADH dehydrogenase [ubiquinone] flavoprotein 2; NDUFV3, NADH dehydrogenase [ubiquinone] flavoprotein 3; NDUFAF2, NADH dehydrogenase (ubiquinone) 1 alpha subcomplex, assembly factor 2.

^c^Best BLAST-X protein sequence match (lowest E value).

^d^Expectation value.

^e^Codifying sequence.

^f^GenBank accession number.
