# Supplementary material for: Unraveling the Molecular Signatures of Oxidative Phosphorylation to Cope with the Nutritionally Changing Metabolic Capabilities of Liver and Muscle Tissues in Farmed Fish
Source: PLoS One. 2015 Apr 15;10(4):e0122889. doi: 10.1371/journal.pone.0122889 (PMC4398389; doi:10.1371/journal.pone.0122889)
Supplement: S2 Table — (DOCX) [file pone.0122889.s002.docx]

**Supporting information Table S2**. **Characteristics of the new gilthead sea bream assembled sequences of Complex II**. Nuclear-encoded catalytic subunits are in red. Nuclear-encoded regulatory subunits are in black. Nuclear-encoded assembly factors are in blue and italics.

| Contigs | F^a^ | Size (nt) | Annotation^b^ | Best match^c^ | E^d^ | CDS^e^ | Accession No^f^ |
| --- | --- | --- | --- | --- | --- | --- | --- |
| C2_1571 | 314 | 3316 | SDHA | XP_003443735 | 0 | 23-2014 | KC217615 |
| C2_791 | 529 | 1381 | SDHB | ACQ58642 | 0 | 47-898 | KC217616 |
| C2_4148 | 125 | 711 | SDHC | XP_003447506 | 8e-99 | 34-546 | KC217617 |
| C2_628 | 560 | 1275 | SDHD | XP_003457657 | 6e-94 | 46-522 | KC217618 |
| C2_4511 | 107 | 1187 | *SDHAF1* | XP_003455545 | 1e-41 | 697-945 | KC217619 |
| C2_1531 | 318 | 1211 | *SDHAF2* | XP_003442349 | 5e-95 | 159-650 | KC217620 |

^a^Number of reads composing the assembled sequences.

^b^Gene identity determined through BLAST searches: SDHA, Succinate dehydrogenase [ubiquinone] flavoprotein subunit; SDHB, Succinate dehydrogenase [ubiquinone] iron-sulfur subunit ; SDHC, Succinate dehydrogenase cytochrome b560 subunit; SDHD, Succinate dehydrogenase [ubiquinone] cytochrome b small subunit B; SDHAF1, Succinate dehydrogenase assembly factor 1; SDHAF2, Succinate dehydrogenase assembly factor 2.

^c^Best BLAST-X protein sequence match (lowest E value).

^d^Expectation value.

^e^Codifying sequence.

^f^GenBank accession number.
