# Supplementary material for: Unraveling the Molecular Signatures of Oxidative Phosphorylation to Cope with the Nutritionally Changing Metabolic Capabilities of Liver and Muscle Tissues in Farmed Fish
Source: PLoS One. 2015 Apr 15;10(4):e0122889. doi: 10.1371/journal.pone.0122889 (PMC4398389; doi:10.1371/journal.pone.0122889)
Supplement: S3 Table — (DOCX) [file pone.0122889.s003.docx]

**Supporting information Table S3**. **Characteristics of the new gilthead sea bream assembled sequences of Complex III**. Nuclear-encoded catalytic subunits are in red. Nuclear-encoded regulatory subunits are in black. Nuclear-encoded assembly factors are in blue and italics.

| Contigs | F^a^ | Size (nt) | Annotation^b^ | Best match^c^ | E^d^ | CDS^e^ | Accession No^f^ |
| --- | --- | --- | --- | --- | --- | --- | --- |
| C2_785 | 309 | 1600 | Cyc1 | XP_003448417 | 0 | 98-1021 | KC217621 |
| C2_507 | 523 | 1386 | UQCRFS1 | XP_003460177 | 3e-160 | 74-895 | KC217622 |
| C2_1152 | 599 | 1960 | UQCRC1 | CBN81058 | 0 | 43-1479 | KC217623 |
| C2_633 | 410 | 1745 | UQCRC2 | CBN81230 | 0 | 73-1452 | KC217624 |
| C2_2118 | 238 | 841 | UQCRH | ACM9291 | 2e-35 | 140-466 | KC217625 |
| C2_516 | 324 | 513 | UQCRB | CAF99385 | 7e-52 | 42-374 | KC217626 |
| C2_12420 | 124 | 526 | UQCRQ | ACQ58134 | 1e-51 | 98-346 | KC217627 |
| C2_1166 | 168 | 564 | UQCR10 | ACQ58386 | 7e-33 | 71-262 | KC217628 |
| C2_2080 | 113 | 523 | UQCR11-A | ACQ582208 | 1e-27 | 88-267 | KC217629 |
| C2_2411 | 201 | 677 | UQCR11-B | CAG07741 | 7e-27 | 113-283 | KC217630 |
| C2_2189 | 191 | 1662 | CYCS | ACQ58095 | 1e-62 | 125-439 | KC217632 |
| C2_1918 | 180 | 1862 | *UQCC* | XP_003456273 | 4e-174 | 138-995 | KC217631 |

^a^Number of reads composing the assembled sequences.

^b^Gene identity determined through BLAST searches: Cyc1, Cytochrome c1, heme protein; UQCRFS1, Cytochrome b-c1 complex subunit Rieske; UQCRC1, Cytochrome b-c1 complex subunit 1; UQCRC2, Cytochrome b-c1 complex subunit 2; UQCRH, Cytochrome b-c1 complex subunit 6; UQCRB, Cytochrome b-c1 complex subunit 7; UQCRQ, Cytochrome b-c1 complex subunit 8; UQCR10, Cytochrome b-c1 complex subunit 9; UQCR11-A, Cytochrome b-c1 complex subunit 10 isoform A; UQCR11-B, Cytochrome b-c1 complex subunit 10 isoform B; UQCC, Ubiquinol-cytochrome c reductase complex chaperone CBP3 homolog; CYCS, Cytochrome c.

^c^Best BLAST-X protein sequence match (lowest E value).

^d^Expectation value.

^e^Codifying sequence.

^f^GenBank accession number.
