# Supplementary material for: Unraveling the Molecular Signatures of Oxidative Phosphorylation to Cope with the Nutritionally Changing Metabolic Capabilities of Liver and Muscle Tissues in Farmed Fish
Source: PLoS One. 2015 Apr 15;10(4):e0122889. doi: 10.1371/journal.pone.0122889 (PMC4398389; doi:10.1371/journal.pone.0122889)
Supplement: S4 Table — (DOCX) [file pone.0122889.s004.docx]

**Supporting information Table S4**. **Characteristics of the new gilthead sea bream assembled sequences of Complex IV**. Mitochondrial-encoded catalytic subunits are in bold and red. Nuclear-encoded regulatory subunits are in black. Nuclear-encoded assembly factors are in blue and italics.

| Contigs | F^a^ | Size (nt) | Annotation^b^ | Best match^c^ | E^d^ | CDS^e^ | Accession No^f^ |
| --- | --- | --- | --- | --- | --- | --- | --- |
| C2_5 | 408 | 1809 | **COXI** | YP_001256941 | 0 | 1-1566 | KC217652 |
| C2_80 | 1718 | 722 | **COXII** | YP_001256942 | 1e-124 | 36-722 | KC217653 |
| C2_5715 | 799 | 783 | **COXIII** | YP_001256945 | 3e-151 | 1-783 | KC217654 |
| C2_3631 | 147 | 696 | NDUFA4 | AEM37711 | 2e-47 | 128-376 | KC217565 |
| C2_462 | 742 | 935 | COX4b | P80971 | 7e-90 | 131-661 | KC217633 |
| C2_238 | 553 | 1195 | COX5a1 | XP_003443919 | 2e-88 | 114-539 | KC217634 |
| C2_745 | 287 | 655 | COX5a2 | XP_003437607 | 2e-92 | 80-505 | KC217635 |
| C2_498 | 458 | 635 | COX5b1 | XP_003437607 | 6e-68 | 105-491 | KC217636 |
| C2_7644 | 41 | 845 | COX5b2 | XP_003441102 | 1e-68 | 30-410 | KC217637 |
| C2_16418 | 28 | 586 | COX6a1 | XP_003445626 | 7e-48 | 22-357 | KC217638 |
| C2_132 | 590 | 607 | COX6a2 | AF051370 | 2e-52 | 177-476 | KC217639 |
| C2_737 | 427 | 603 | COX6b1a | ACQ58800 | 1e-53 | 88-348 | KC217640 |
| C2_1197 | 147 | 532 | COX6b1b | [CBN81268](http://www.ncbi.nlm.nih.gov/protein/317419231?report=genbank&log$=prottop&blast_rank=1&RID=YKBC7YM701N) | 1e-59 | 78-338 | KC217641 |
| C2_2923 | 122 | 501 | COX6c1 | P80977 | 2e-27 | 117-347 | KC217642 |
| C2_5899 | 107 | 508 | COX7a1 | XP_003448990 | 1e-39 | 99-344 | KC217643 |
| C2_1148 | 223 | 517 | COX7a2 | CAG01459 | 5e-42 | 72-323 | KC217644 |
| C2_3512 | 123 | 489 | COX7b | ACO09254 | 2e-38 | 84-326 | KC217645 |
| C2_24926 | 24 | 389 | COX7c | ADG29167 | 1e-35 | 64-255 | KC217646 |
| C2_3031 | 80 | 518 | COX8a | XP_003458202 | 2e-25 | 67-279 | KC217647 |
| C2_203 | 548 | 1045 | COX8b | AER42691 | 2e-24 | 254-457 | KC217648 |
| C2_6516 | 101 | 1371 | *SCO1* | ACQ59007 | 2e-155 | 88-1008 | KC217649 |
| C2_5469 | 93 | 1150 | *SURF1* | NP_001135069 | 8e-163 | 100-1017 | KC217650 |
| C2_2595 | 220 | 2114 | *COX15* | XP_003441034 | 0 | 139-1362 | KC217651 |

^a^Number of reads composing the assembled sequences.

^b^Gene identity determined through BLAST searches: COXI, Cytochrome c oxidase subunit 1; COXII, Cytochrome c oxidase subunit 2; COXIII, Cytochrome c oxidase subunit 3; NDUFA4, NADH dehydrogenase [ubiquinone] 1 alpha subcomplex subunit 4; COX4b, Cytochrome c oxidase subunit 4 isoform 2; COX5a1, Cytochrome c oxidase subunit 5A isoform 1; COX5a2, Cytochrome c oxidase subunit 5A isoform 2; COX5b1, Cytochrome c oxidase subunit 5B isoform 1; COX5b2, Cytochrome c oxidase subunit 5B isoform 2; COX6a1, Cytochrome c oxidase subunit 6A isoform 1; COX6a2, Cytochrome c oxidase subunit 6A isoform 2; COX6b1a, Cytochrome c oxidase subunit VIb isoform 1a; COX6b1b, Cytochrome c oxidase subunit VIb isoform 1b ; COX6c1, Cytochrome c oxidase subunit 6C-1; COX7a1, Cytochrome c oxidase subunit 7A1; COX7a2, Cytochrome c oxidase subunit 7A2; COX7b, Cytochrome c oxidase subunit 7B; COX7c, Cytochrome c oxidase subunit 7C; COX8a, Cytochrome c oxidase subunit 8A; COX8b, Cytochrome c oxidase subunit 8B; COX15, Cytochrome c oxidase assembly protein COX15 homolog; SCO1, SCO1 protein homolog; SURF1, Surfeit locus protein 1.

^c^Best BLAST-X protein sequence match (lowest E value).

^d^Expectation value.

^e^Codifying sequence.

^f^GenBank accession number.
