# Supplementary material for: Unraveling the Molecular Signatures of Oxidative Phosphorylation to Cope with the Nutritionally Changing Metabolic Capabilities of Liver and Muscle Tissues in Farmed Fish
Source: PLoS One. 2015 Apr 15;10(4):e0122889. doi: 10.1371/journal.pone.0122889 (PMC4398389; doi:10.1371/journal.pone.0122889)
Supplement: S5 Table — (DOCX) [file pone.0122889.s005.docx]

**Supporting information Table S5**. **Characteristics of the new gilthead sea bream assembled sequences of Complex V**. Mitochondrial-encoded catalytic subunits are in bold and red. Nuclear-encoded catalytic subunits are in red. Nuclear-encoded regulatory subunits are in black. Nuclear-encoded assembly factors are in blue and italics.

| Contigs | F^a^ | Size (nt) | Annotation^b^ | Best match^c^ | E^d^ | CDS^e^ | Accession No^f^ |
| --- | --- | --- | --- | --- | --- | --- | --- |
| C3_39963 | 134 | 671 | **ATP6** | YP_001256944 | 9e-45 | 273->671 | KC217599 |
| C3_114147 | 10 | 214 | **ATP8** | YP_001256943 | 3e-22 | 1-165 | KC217600 |
| C2_1751 | 446 | 1891 | ATP5A1 | CAF96443 | 0 | 88-1743 | KC217601 |
| C2_1973 | 282 | 1782 | ATP5B | XP_003447376 | 0 | 68-1624 | KC217602 |
| C2_18579 | 502 | 1302 | ATP5C1 | XP_003448120 | 0 | 50-931 | KC217603 |
| C2_6419 | 109 | 709 | ATP5D | CAF92415 | 2e-94 | 55-549 | KC217604 |
| C2_24277 | 85 | 409 | ATP5E | XP_003444817 | 3e-20 | 61-219 | KC217605 |
| C2_176 | 883 | 1091 | ATP5F1 | ACQ58228 | 1e-146 | 150-902 | KC217606 |
| C2_107 | 762 | 1116 | ATP5G1 | ADG29151 | 6e-69 | 233-652 | KC217607 |
| C2_6236 | 132 | 586 | ATP5H | XP_003450004 | 2e-94 | 51-536 | KC217608 |
| C2_7051 | 97 | 901 | ATP5I | CAG12583 | 6e-17 | 85-300 | KC217609 |
| C2_1188 | 233 | 546 | ATP5J2 | CAF95291 | 7e-51 | 47-316 | KC217610 |
| C2_1627 | 245 | 506 | ATP5L | CAF94261 | 6e-58 | 91-402 | KC217611 |
| C2_123 | 968 | 969 | ATPO | CAF99056 | 1e-122 | 187-816 | KC217612 |
| C2_19166 | 23 | 1381 | OSCP1 | XP_003450561 | 0 | <1-1030 | KC217613 |
| C2_2673 | 262 | 1925 | *ATPAF2* | CBN81217 | 5e-177 | 289-1146 | KC217614 |

^a^ Number of reads composing the assembled sequences.

^b^ Gene identity determined through BLAST searches: ATP6, ATP synthase subunit a; ATP8, ATP synthase protein 8; ATP5A1, ATP synthase subunit alpha; ATP5B, ATP synthase subunit beta; ATP5C1, ATP synthase subunit gamma; ATP5D, ATP synthase subunit delta; ATP5E, ATP synthase subunit epsilon; ATP5F1, ATP synthase subunit b; ATP5G1, ATP synthase lipid-binding protein; ATP5H, ATP synthase subunit d; ATP5I, ATP synthase subunit e; ATP5J2, ATP synthase subunit f; ATP5L, ATP synthase subunit g; ATP5O, ATP synthase subunit O; OSCP1, Protein OSCP1; ATPAF2, ATP synthase mitochondrial F1 complex assembly factor 2.

^c^ Best BLAST-X protein sequence match (lowest E value).

^d^ Expectation value.

^e^ Codifying domain sequence.

^f^GenBank accession number.
