# Supplementary material for: Unraveling the Molecular Signatures of Oxidative Phosphorylation to Cope with the Nutritionally Changing Metabolic Capabilities of Liver and Muscle Tissues in Farmed Fish
Source: PLoS One. 2015 Apr 15;10(4):e0122889. doi: 10.1371/journal.pone.0122889 (PMC4398389; doi:10.1371/journal.pone.0122889)
Supplement: S6 Table — (DOCX) [file pone.0122889.s006.docx]

**Supporting information Table S6.** **Forward and reverse primers for real-time PCR of Complex I.** Mitochondrial-encoded catalytic subunits are in bold and red. Nuclear-encoded catalytic subunits are in red. Nuclear-encoded regulatory subunits are in black. Nuclear-encoded assembly factors are in blue and italics.

| Gene name | Symbol |  | Primer sequence |
| --- | --- | --- | --- |
| NADH-ubiquinone oxidoreductase chain 2 | **ND2** | F | TAG GTT GAA TGA CCA TCG TA |
|  |  | R | GGC TAA GGA GTT GAG GTT |
| NADH-ubiquinone oxidoreductase chain 5 | **ND5** | F | CCT AAA CGC CTG AGC CCT GG |
|  |  | R | GCT GTA AAC GAG GTG GCT AGA AGG |
| NADH dehydrogenase [ubiquinone] 1 alpha subcomplex subunit 1 | NDUFA1 | F | CGG GTT CCG TGG CAG TGG TA |
|  |  | R | TCC TGT TCC TGA TAC TCG CTT GTC TCT |
|  |  |  |  |
| NADH dehydrogenase [ubiquinone] 1 alpha subcomplex subunit 2 | NDUFA2 | F | CCA CCT CTG CCA GAC CTC |
|  |  | R | ACT CAC ATA GTG CTG CTC CA |
|  |  |  |  |
| NADH dehydrogenase [ubiquinone] 1 alpha subcomplex subunit 3 | NDUFA3 | F | TCG GAG CGT TCC TGA AGA ATG C |
|  |  | R | GAA GAG CCA TAC CTA TCA GTC CAA TAC CA |
|  |  |  |  |
| NADH dehydrogenase [ubiquinone] 1 alpha subcomplex subunit 4-like 2 | NDUFA4-like2 | F | TCG GAC AGC AGT AGA GCA T |
|  |  | R | CGC CCA TTC CCA AAC AGA T |
|  |  |  |  |
| NADH dehydrogenase [ubiquinone] 1 alpha subcomplex subunit 5 | NDUFA5 | F | ATG GCT GGC TTG CTG AAA |
|  |  | R | AGA CGC TCA TGT GGA TTG TT |
|  |  |  |  |
| NADH dehydrogenase [ubiquinone] 1 alpha subcomplex subunit 6 | NDUFA6 | F | TCA GGG AAG GGA CAA AGT GAG GGA GAT G |
|  |  | R | CGT GGG TCG GTG ACA TGC TTG TTC TT |
|  |  |  |  |
| NADH dehydrogenase [ubiquinone] 1 alpha subcomplex subunit 7 | NDUFA7 | F | CCG AGC CAC AAG TAT GCC AGC AAC TA |
|  |  | R | AGC CTC CCT GCG TCC ATC TCT G |
|  |  |  |  |
| NADH dehydrogenase [ubiquinone] 1 alpha subcomplex subunit 8 | NDUFA8 | F | GCT CCC AGT GTG ACA AAC CCA ACA AAG A |
|  |  | R | CCT TCT CCT CCC AGC GGC AGA G |
|  |  |  |  |
| NADH dehydrogenase [ubiquinone] 1 alpha subcomplex subunit 9 | NDUFA9 | F | GGC TGC TCT CCT GCT GTG TT |
|  |  | R | CCT CTG CTG GAC TGT GGT GAC |
|  |  |  |  |
| NADH dehydrogenase [ubiquinone] 1 alpha subcomplex subunit 12 | NDUFA12 | F | TGG CGG AGT ATG CGA ACC T |
|  |  | R | CTC GGA CTC CAC CAT GAC CT |
|  |  |  |  |
| NADH dehydrogenase [ubiquinone] 1 beta subcomplex subunit 1 | NDUFB1 | F | CCG TGA GCA TTG GGT GAA CAT CTT |
|  |  | R | TTC TGG TCC TGC TGT TTG TCA AGG T |
|  |  |  |  |
| NADH dehydrogenase [ubiquinone] 1 beta subcomplex subunit 2 | NDUFB2 | F | CAG AGG ATA ACG ACC AGA AAG GC |
|  |  | R | CCC TGT ACT GTG GCT CAA TGT G |
|  |  |  |  |
| NADH dehydrogenase [ubiquinone] 1 beta subcomplex subunit 3 | NDUFB3 | F | GGA ACG AGG CAT GGA GAT AC |
|  |  | R | CTA CAG CCA GAG CAA CAG TAA |

**Supp. Table 6.** Continued.

| Gene name | Symbol |  | Primer sequence |
| --- | --- | --- | --- |
| NADH dehydrogenase [ubiquinone] 1 beta subcomplex subunit 4 | NDUFB4 | F | TCG TGG GTC TGC TGT TTG GAG TTG TG |
|  |  | R | ATC TGT GCC TCC TTC CTA TCC CTG TCT GT |
|  |  |  |  |
| NADH dehydrogenase [ubiquinone] 1 beta subcomplex subunit 5 | NDUFB5 | F | TGC GTC GGC AGA TGA GGA T |
|  |  | R | CTT GTT GAG GGT GTT CAC CTG GAA |
|  |  |  |  |
| NADH dehydrogenase [ubiquinone] 1 beta subcomplex subunit 6 | NDUFB6 | F | GAG CCC AAG AGC CTG TGG AGA CT |
|  |  | R | CAG GTA GCA GGA GGC GTG TTA ATG TGA A |
|  |  |  |  |
| NADH dehydrogenase [ubiquinone] 1 beta subcomplex subunit 8 | NDUFB8 | F | AAG GGA AGG GCT CTG GCA TTT |
|  |  | R | GGC GGT CCA AGT ACA GTA TCC T |
|  |  |  |  |
| NADH dehydrogenase [ubiquinone] 1 beta subcomplex subunit 9 | NDUFB9 | F | CAA AGA CCG AAG CCC TCC |
|  |  | R | TGA CAT ACT GCC ACC ACA A |
|  |  |  |  |
| NADH dehydrogenase [ubiquinone] 1 beta subcomplex subunit 10 | NDUFB10 | F | ACT GTG CCC ACG AAC TGA A |
|  |  | R | ATG CTC CCA GGT CTC CAT AG |
|  |  |  |  |
| NADH dehydrogenase [ubiquinone] 1 beta subcomplex subunit 11 | NDUFB11 | F | GTG ACC CTG TGG TGG ATG AGT GGA A |
|  |  | R | ACT GAG ATG CCG AAG AAG AAG CCA ACC T |
|  |  |  |  |
| NADH dehydrogenase 1 subunit C1 | NDUFC1 | F | GCA CTC GTC AGC AGA GTT GGA T |
|  |  | R | GTT GGC AGT GTC AGG CTT GGA |
| NADH dehydrogenase 1 subunit C2 | NDUFC2 | F | CTT CAG AAT GCC ATC AAC CAC AG |
|  |  | R | CCG AGG AAC CAG CCA ACT |
| NADH dehydrogenase iron-sulfur protein 2 | NDUFS2 | F | GTA TCA GAC GGC TCC AGC AGA C |
|  |  | R | AGA CCA GCC AAG TGA GCG AAT |
| NADH dehydrogenase iron-sulfur protein 4 | NDUFS4 | F | AGC CAA CAC CAA GAA GTG GAA GA |
|  |  | R | AAG CCC AGC CCA TCA GAG G |
| NADH dehydrogenase iron-sulfur protein 5 | NDUFS5 | F | CAG CAG CGT GAC AAG ATG GTG AA |
|  |  | R | CAG GGC GGT GGC GTG TAG |
| NADH dehydrogenase iron-sulfur protein 6 | NDUFS6 | F | GCC GTA CCT GTT CAT CGT TA |
|  |  | R | TTC GCA TCG TAG ACC TGT C |
| NADH dehydrogenase iron-sulfur protein 7 | NDUFS7 | F | AAC GGA GGA GGC TAC TAC CAC TAC T |
|  |  | R | CGG TAC GAT TCG GTC ACA ACC TCT AAC |
| NADH dehydrogenase [ubiquinone] flavoprotein 1 | NDUFV1 | F | CGT GCT CCA GTT GCT GTC AG |
|  |  | R | TGT GTG CTG TTG AAT CGG GTG ATA |
| NADH dehydrogenase [ubiquinone] flavoprotein 2 | NDUFV2 | F | CAG GCA GGT CAG GAA TCT |
|  |  | R | CAG CAT TGT TGT CAG GAG TAT |
| NADH dehydrogenase [ubiquinone] flavoprotein 3 | NDUFV3 | F | ACT GCG AGC ACC ACA CCT ACA AC |
|  |  | R | CAC GTC CAG GTC GGC GAA TGT |
| NADH dehydrogenase (ubiquinone) 1 alpha subcomplex, assembly factor 2 | *NDUFAF2* | F | AGG CAG CAT ACC GAT AGA G |
|  |  | R | ACT CAT TCT TCA GCA ACT CCT |
|  |  |  |  |
|  |  |  |  |
