# Supplementary material for: Unraveling the Molecular Signatures of Oxidative Phosphorylation to Cope with the Nutritionally Changing Metabolic Capabilities of Liver and Muscle Tissues in Farmed Fish
Source: PLoS One. 2015 Apr 15;10(4):e0122889. doi: 10.1371/journal.pone.0122889 (PMC4398389; doi:10.1371/journal.pone.0122889)
Supplement: S7 Table — (DOC) [file pone.0122889.s007.doc]

**Supporting information Table S7.** **Forward and reverse primers for real-time PCR of Complex II.** Nuclear-encoded catalytic subunits are in red. Nuclear-encoded regulatory subunits are in black. Nuclear-encoded assembly factors are in blue and italics.

| Gene name | Symbol |  | Primer sequence |
| --- | --- | --- | --- |
| Succinate dehydrogenase [ubiquinone] flavoprotein subunit | SDHA | F | CAA TCT CTG GAT GAG CAG GAC TGT |
|  |  | R | GTA GGA GCG GAT GGC AGG AG |
|  |  |  |  |
| Succinate dehydrogenase [ubiquinone] iron-sulfur subunit | SDHB | F | TGG TGG CGG TGC GGT ATG |
|  |  | R | ATT CTG GGT TGT GCT GCT GGA G |
|  |  |  |  |
| Succinate dehydrogenase cytochrome b560 subunit | SDHC | F | AGT GAC ACA CAG AGG AAC TGG AGT |
|  |  | R | CAG GGC AAA GGC GGA GAT AGC |
| Succinate dehydrogenase [ubiquinone] cytochrome b small subunit B | SDHD | F | CAT CAG GAG CAG CCG TAC A |
|  |  | R | CCA GAG GCA GCG TAC AGA G |
|  |  |  |  |
| Succinate dehydrogenase assembly factor 1 | *SDHAF1* | F | CCA GGA CAA ACC AGG CTT CAT C |
|  |  | R | GTC TTC TTG ATG CGA GCG TTC T |
| Succinate dehydrogenase assembly factor 2 | *SDHAF2* | F | CCA GCG AGT CCA TTG ACA TCA |
|  |  | R | CCC TCT TTC GGC TCT CAT ACA G |
