# Supplementary material for: Unraveling the Molecular Signatures of Oxidative Phosphorylation to Cope with the Nutritionally Changing Metabolic Capabilities of Liver and Muscle Tissues in Farmed Fish
Source: PLoS One. 2015 Apr 15;10(4):e0122889. doi: 10.1371/journal.pone.0122889 (PMC4398389; doi:10.1371/journal.pone.0122889)
Supplement: S8 Table — (DOCX) [file pone.0122889.s008.docx]

**Supporting information Table S8. Forward and reverse primers for real-time PCR of Complex III.** Mitochondrial-encoded catalytic subunits are in bold and red. Nuclear-encoded catalytic subunits are in red. Nuclear-encoded regulatory subunits are in black. Nuclear-encoded assembly factors are in blue and italics.

| Gene name | Symbol |  | Primer sequence |
| --- | --- | --- | --- |
| Cytochrome b | **CYB** | F | TGA CAG GGC TAT TCC TCG CTA TGC |
|  |  | R | AGA TGT GGG CTA CGG AAG AGA AGG |
| Cytochrome c | CYCS | F | CAT ACA CTG ACG CCA ACA AGA GTA AAG GT |
|  |  | R | GGG TTC TCC AGG TAC TCC ATC AAG GT |
| Cytochrome c1, heme protein, mitochondrial | Cyc1 | F | GCT GAG GAG GTG GAG GTT GT |
|  |  | R | CCT GGA CGG GTG AAC ATC TCT |
| Cytochrome b-c1 complex subunit Rieske, mitochondrial | UQCRFS1 | F | GCG ACT TCG GTG GTT ACT ACT G |
|  |  | R | GCG ACC TGA GGC GTC ATA ATG |
|  |  |  |  |
| Cytochrome b-c1 complex subunit 1, mitochondrial | UQCRC1 | F | TGT CCT GCT GTG GTT GCT GTT |
|  |  | R | CGC ACT CTG TTG TAG TCG GGT AG |
| Cytochrome b-c1 complex subunit 2 | UQCRC2 | F | GAG CAA TTC CTC AAC ATT CG |
|  |  | R | TCT CAC CTC CAC GAT ACT G |
| Cytochrome b-c1 complex subunit 6 | UQCRH | F | AAG TGT GAG CAG ACT GAA C |
|  |  | R | TCA GTG TGG GAT CTG GAG |
| Cytochrome b-c1 complex subunit 7 | UQCRB | F | CGA CAG GAC CTT CAG GAT G |
|  |  | R | CTC TCA CGG ACC ACC TCA T |
| Cytochrome b-c1 complex subunit 8 | UQCRQ | F | GTC AGA CAT ATT ATC ACC TA |
|  |  | R | GAT TCC CTT TGA GAA GTA |
|  |  |  |  |
| Cytochrome b-c1 complex subunit 9 | UQCR10 | F | GCT GGC GAA GTC CGT CTA CAA |
|  |  | R | AGA GAA CCG CTC CAA CCA TGA TG |
| Cytochrome b-c1 complex subunit 10 isoform A | UQCR11-A | F | CTA TTC TGA GGG CGT GGG |
|  |  | R | TCT GTG AAG TAG ACG AGC G |
| Cytochrome b-c1 complex subunit 10 isoform B | UQCR11-B | F | GGG CTC AGT GGG AGG TGT A |
|  |  | R | ACA ATC GCC AGT CTG TGA AGT G |
| Ubiquinol-cytochrome c reductase complex chaperone CBP3 homolog | *UQCC* | F | CAG TCC TTC GTC AGA ACC GCT TT |
|  |  | R | GCA GGC TTC TTG ATC TAA CAC CTT TCC |
|  |  |  |  |
