# Supplementary material for: Unraveling the Molecular Signatures of Oxidative Phosphorylation to Cope with the Nutritionally Changing Metabolic Capabilities of Liver and Muscle Tissues in Farmed Fish
Source: PLoS One. 2015 Apr 15;10(4):e0122889. doi: 10.1371/journal.pone.0122889 (PMC4398389; doi:10.1371/journal.pone.0122889)
Supplement: S9 Table — (DOCX) [file pone.0122889.s009.docx]

**Supporting information Table S9.** **Forward and reverse primers for real-time PCR of Complex IV.** Mitochondrial-encoded catalytic subunits are in bold and red. Nuclear-encoded regulatory subunits are in black. Nuclear-encoded assembly factors are in blue and italics.

| Gene name | Symbol |  | Primer sequence |
| --- | --- | --- | --- |
| Cytochrome c oxidase subunit 1 | **COXI** | F | GTC CTA CTT CTT CTG TCC CTT CCT GTT CT |
|  |  | R | AGG TTT CGG TCT GTA AGG AGC ATT GTA ATC |
| Cytochrome c oxidase subunit 2 | **COXII** | F | ACT GCC TAC ACA GGA CCT TGC C |
|  |  | R | GTC TGC TTC CAG GAG ACG GAA TTG T |
| Cytochrome c oxidase subunit 3 | **COXIII** | F | CCA AGC ACA CGC ATA CCA CAT A |
|  |  | R | GCG GCA ACT GCA CCT GTA |
| Cytochrome c oxidase subunit 4 isoform 2, mitochondrial | NDUFA4 | F | TCG TGT GGT GGC AGA GCA TCT AC |
|  |  | R | CGA AGG TCC TGG GGC GTT CA |
|  |  |  |  |
| NADH dehydrogenase [ubiquinone] 1 alpha subcomplex subunit 4 | COX4b | F | GCT CGT CTG GGC TTG AGA AAC C |
|  |  | R | GCT CTG GGT TGT TCT TGC GAT CC |
| Cytochrome c oxidase subunit 5A, mitochondrial-like isoform 1 | COX5a1 | F | CAC GCA ACC ACG GTG CCA AG |
|  |  | R | TGT CTC CTG CTT GCC ATG TGA GTA AC |
|  |  |  |  |
| Cytochrome c oxidase subunit 5A, mitochondrial-like isoform 2 | COX5a2 | F | CGC CAT CCG CAT CCT TGA |
|  |  | R | GGC TTC AAC TCT TGG ATC AGG TAGG |
|  |  |  |  |
| Cytochrome c oxidase subunit 5B isoform 1, mitochondrial | COX5b1 | F | GCC AGA CCG TTA CAC CGT GCT AT |
|  |  | R | CCT GTT CCT CAT CTG TTG GTA TTC CTC TCA |
|  |  |  |  |
| Cytochrome c oxidase subunit 5B isoform 2, mitochondrial | COX5b2 | F | GAT GAA GCC GAA GGA GTA CG |
|  |  | R | GAT GGA GGG AAC GAG GTG |
|  |  |  |  |
| Cytochrome c oxidase subunit 6A isoform 1, mitochondrial | COX6a1 | F | GAG CAG AGT CAC AGC CAC GAG |
|  |  | R | TTG CTG CGA ATG CGA AGA TGG |
| Cytochrome c oxidase subunit 6A isoform 2, mitochondrial | COX6a2 | F | TGT TGG CTG CTG CGT CAC ATT C |
|  |  | R | CAG AAT CTT CCA GGT CCT CGC TCC |
| Cytochrome c oxidase subunit VIb isoform 1a | COX6b1a | F | TCA GCC AGA GAC CAG GAC AC |
|  |  | R | GGG CAG AGG CTC TTG TAA ACC |
| Cytochrome c oxidase subunit VIb isoform 1b | COX6b1b | F | CCA ACC AGA ACC AGA CCA GGA ACT |
|  |  | R | GGC AGC GGT GAT AGT CCA GGT AG |
| Cytochrome c oxidase subunit 6C-1 | COX6c1 | F | TCT CTC TGT CAC TCC TGG CTG CGA TAG |
|  |  | R | CCT GGG CTC TGT CAC TGC GTA CTT G |
| Cytochrome c oxidase subunit 7A1 | COX7a1 | F | GGC GAC CAT GAC GTT AAC CAT CAC AGG AA |
|  |  | R | GGT GAG CAG CCA GTA GCA GGA GTA ACA |
| Cytochrome c oxidase subunit 7A2 | COX7a2 | F | AAG GAG GCA GCA GCG ATG |
|  |  | R | TCC AGC ACC GAG GAC AGT |
| Cytochrome c oxidase subunit 7B | COX7b | F | TCT TCT GTG TGG CTG TGT GGT CAT ACG |
|  |  | R | TTC CCA ACA GGT GAC AAA TTC CAG GTG AT |
| Cytochrome c oxidase subunit 7C | COX7c | F | GGT GGT GTT CTT TGG CAG TGG CTT T |
|  |  | R | CAG GAT CTG ATG TCT GAC GAC GAT GAA GG |
| Cytochrome c oxidase subunit 8A | COX8a | F | GCA GAC CGC CCA AAG ACG |
|  |  | R | CAT GCC AAT TCC GAC GAC AGT T |
| Cytochrome c oxidase subunit 8B, mitochondrial | COX8b | F | TCC GCT GGT CCC TGT GGC TAA |
|  |  | R | CCT CCA CTG ATA TTG TGT TTG GCA GGT TTG |

**Supp. Table 9.** Continued.

| Gene name | Symbol |  | Primer sequence |
| --- | --- | --- | --- |
| SCO1 protein homolog, mitochondrial | *SCO1* | F | ACA ACA ACA AGC CCA CCA AGA |
|  |  | R | GAC AGT GAG TGA ACC CGA AGT AGA T |
| Surfeit locus protein 1 | *SURF1* | F | AGA TGG AAG GTG AAG TGG AGG TGG TC |
|  |  | R | GCG TTG CTC TGT CTG CCG AAC T |
| Cytochrome c oxidase assembly protein COX15 homolog | *COX15* | F | CAT ACT AGG TCG CTG GTT AG |
|  |  | R | GAT TCC GTG AGC CTT GTG |
|  |  |  |  |
