# Supplementary material for: Unraveling the Molecular Signatures of Oxidative Phosphorylation to Cope with the Nutritionally Changing Metabolic Capabilities of Liver and Muscle Tissues in Farmed Fish
Source: PLoS One. 2015 Apr 15;10(4):e0122889. doi: 10.1371/journal.pone.0122889 (PMC4398389; doi:10.1371/journal.pone.0122889)
Supplement: S10 Table — (DOCX) [file pone.0122889.s010.docx]

**Supporting information Table S10.** **Forward and reverse primers for real-time PCR of Complex V.** Nuclear-encoded catalytic subunits are in red. Nuclear-encoded regulatory subunits are in black. Nuclear-encoded assembly factors are in blue and italics.

| Gene name | Symbol |  | Primer sequence |
| --- | --- | --- | --- |
| ATP synthase subunit alpha, mitochondrial | ATP5A1 | F | CGT CAT CTC CAT CAC AGA CGG ACA GA |
|  |  | R | CGA ATA CCC TTG TAG AAC AGC TCA GTC TCC |
| ATP synthase subunit beta, mitochondrial | ATP5B | F | GTA GCA CTG GTG TAT GGT CAG ATG AAC GA |
|  |  | R | TCT GGC ACG GGC ACC TGG |
| ATP synthase subunit gamma | ATP5C1 | F | GAG GAG AAG GTC GCC AAG CAT |
|  |  | R | GCC ACA GAG ACC ACG ATC AGA G |
| ATP synthase subunit delta | ATP5D | F | TGC CCA TGT ACC CAC CCT |
|  |  | R | ACA GCG GAA CCG TCA TCA TT |
| ATP synthase subunit epsilon | ATP5E | F | TGG TCG CAT ACT GGA GAC AAG CA |
|  |  | R | GCA GAT AGA GGA GAA GCG GAT GTA GC |
| ATP synthase subunit b | ATP5F1 | F | CCG CAG GAT GGA GCA GGA G |
|  |  | R | TGC CGA CGA CGC TCT TCT C |
| ATP synthase lipid-binding protein | ATP5G1 | F | GGA GTC GCT GGA TCT GGA GCT GG TAT T |
|  |  | R | ATA GCC AAT GAT GAG ACT GCC GAA CAC TGT |
| ATP synthase subunit e | ATP5I | F | CTC CAG TTG CAG TGT CGC CTC TGA TTA |
|  |  | R | TCT CCT TTT GCC GTA GAT GAT CCC AGC |
| ATP synthase subunit f | ATP5J2 | F | GTT GCC ATT GTT GAG AAG CGT CTG A |
|  |  | R | CCT CCA AGC CAG GTT CCA AGC |
| ATP synthase subunit g | ATP5L | F | ACC TTC ACC TGC CGA GAT |
|  |  | R | CAG CCT GGA AGC TCT TGA TG |
| ATP synthase subunit O | ATP5O | F | GCT ATG CCA CCG CTC TGT TCT C |
|  |  | R | CTG CTC CAC TTG GTC CAG TTT CTT C |
| Protein OSCP1 | OSCP1 | F | CAG CGG TCC TGT GCC TTA TGG |
|  |  | R | GCC TGG TCG TCT CTT CAC CTG TA |
| ATP synthase mitochondrial F1 complex assembly factor 2 | *ATPAF2* | F | CGT TCT GGC GAT GGC GAT GAT TGA CA |
|  |  | R | TGG AGA GCA GCA CAG CCT GTT CTA CG |
