# Supplementary material for: Unraveling the Molecular Signatures of Oxidative Phosphorylation to Cope with the Nutritionally Changing Metabolic Capabilities of Liver and Muscle Tissues in Farmed Fish
Source: PLoS One. 2015 Apr 15;10(4):e0122889. doi: 10.1371/journal.pone.0122889 (PMC4398389; doi:10.1371/journal.pone.0122889)
Supplement: S11 Table — (DOCX) [file pone.0122889.s011.docx]

**Supporting information Table S11**. **Relative gene expression of OXPHOS genes in liver, white skeletal muscle (WSM) and heart of control and fasted fish.** Values are the mean ± SEM (n = 5-7). β-Actin was used as housekeeping gene. NDUFC2, NDUFA5 and NDUFB2 were used as reference genes (arbitrary values of 1) in liver, WM and heart, respectively. Within each tissue, means with asterisk are significantly different from control fish (P<0.05; Student’s *t* test). Mitochondrial-encoded catalytic subunits are in bold and red. Nuclear-encoded catalytic subunits are in red. Nuclear-encoded regulatory subunits are in black. Nuclear-encoded assembly factors are in blue and italics.

| Gene name^+^ | Liver | | |  | WSM | | |  | Heart | | |
| --- | --- | --- | --- | --- | --- | --- | --- | --- | --- | --- | --- |
|  | CTRL |  | Fasted |  | CTRL |  | Fasted |  | CTRL |  | Fasted |
| ND2 | 27.13 ± 3.43 |  | 10 ± 1.41* |  | 11.43 ± 1.26 |  | 11.71 ± 1.56 |  | 24.06 ± 4.21 |  | 22.5 ± 1.36 |
| ND5 | 9.6 ± 0.36 |  | 2.94 ± 0.35* |  | 4.97 ± 0.61 |  | 3.99 ± 0.57 |  | 9.49 ± 0.89 |  | 11.28 ± 0.57 |
| NDUFA1 | 0.91 ± 0.06 |  | 0.47 ± 0.04* |  | 0.83 ± 0.06 |  | 1.13 ± 0.1* |  | 0.94 ± 0.09 |  | 1.21 ± 0.13 |
| NDUFA2 | 0.6 ± 0.08 |  | 0.34 ± 0.04* |  | 0.87 ± 0.11 |  | 0.73 ± 0.1 |  | 1.47 ± 0.14 |  | 1.26 ± 0.16 |
| NDUFA3 | 0.81 ± 0.08 |  | 0.36 ± 0.03* |  | 0.52 ± 0.06 |  | 0.63 ± 0.07 |  | 1.15 ± 0.07 |  | 1.15 ± 0.12 |
| NDUFA4-like2 | 0.29 ± 0.01 |  | 0.14 ± 0.02* |  | 0.13 ± 0.05 |  | 0.06 ± 0.04 |  | 0.003 ± 0.00 |  | 0.002 ± 0.00 |
| NDUFA5 | 1.02 ± 0.08 |  | 0.48 ± 0.04* |  | 1.04 ± 0.16 |  | 1.03 ± 0.08 |  | 1.53 ± 0.13 |  | 1.45 ± 0.12 |
| NDUFA6 | 0.4 ± 0.05 |  | 0.29 ± 0.01* |  | 0.49 ± 0.05 |  | 0.71 ± 0.05* |  | 0.98 ± 0.12 |  | 0.92 ± 0.08 |
| NDUFA7 | 0.25 ± 0.02 |  | 0.17 ± 0.02* |  | 0.24 ± 0.03 |  | 0.42 ± 0.04* |  | 0.51 ± 0.03 |  | 0.61 ± 0.04 |
| NDUFA8 | 0.11 ± 0.01 |  | 0.07 ± 0.01* |  | 0.14 ± 0.01 |  | 0.18 ± 0.02* |  | 0.3 ± 0.04 |  | 0.31 ± 0.03 |
| NDUFA9 | 0.5 ± 0.08 |  | 0.22 ± 0.02* |  | 0.38 ± 0.04 |  | 0.5 ± 0.06 |  | 0.68 ± 0.03 |  | 0.59 ± 0.04 |
| NDUFA12 | 0.52 ± 0.06 |  | 0.27 ± 0.02* |  | 0.43 ± 0.08 |  | 0.43 ± 0.04 |  | 0.89 ± 0.04 |  | 0.92 ± 0.08 |
| NDUFB1 | 0.58 ± 0.13 |  | 0.31 ± 0.02 |  | 0.57 ± 0.06 |  | 0.44 ± 0.04 |  | 1.19 ± 0.11 |  | 1.08 ± 0.13 |
| NDUFB2 | 0.48 ± 0.05 |  | 0.24 ± 0.01* |  | 0.56 ± 0.05 |  | 0.67 ± 0.07 |  | 0.94 ± 0.05 |  | 0.94 ± 0.05 |
| NDUFB3 | 2.08 ± 0.28 |  | 1.46 ± 0.13* |  | 1.34 ± 0.24 |  | 1.43 ± 0.22 |  | 1.97 ± 0.28 |  | 2.04 ± 0.26 |
| NDUFB4 | 0.31 ± 0.07 |  | 0.14 ± 0.02* |  | 0.51 ± 0.04 |  | 0.58 ± 0.08 |  | 1.27 ± 0.07 |  | 0.98 ± 0.07 |
| NDUFB5 | 0.58 ± 0.1 |  | 0.33 ± 0.02* |  | 0.73 ± 0.06 |  | 1.22 ± 0.17* |  | 1.22 ± 0.15 |  | 1.2 ± 0.1 |
| NDUFB6 | 0.25 ± 0.06 |  | 0.14 ± 0.01* |  | 0.34 ± 0.03 |  | 0.34 ± 0.06 |  | 0.92 ± 0.1 |  | 0.73 ± 0.08 |
| NDUFB8 | 1.2 ± 0.21 |  | 0.78 ± 0.09 |  | 1.03 ± 0.11 |  | 1.29 ± 0.2 |  | 1.62 ± 0.22 |  | 1.6 ± 0.15 |
| NDUFB9 | 0.005 ± 0.00 |  | 0.003 ± 0.00* |  | 0.006 ± 0.00 |  | 0.009 ± 0.00* |  | 0.006 ± 0.00 |  | 0.005 ± 0.00 |
| NDUFB10 | 1.07 ± 0.11 |  | 0.55 ± 0.06* |  | 1.01 ± 0.1 |  | 1.46 ± 0.11* |  | 1.47 ± 0.12 |  | 1.22 ± 0.1 |
| NDUFB11 | 0.3 ± 0.04 |  | 0.15 ± 0.01* |  | 0.35 ± 0.04 |  | 0.47 ± 0.06 |  | 0.53 ± 0.04 |  | 0.57 ± 0.03 |
| NDUFC1 | 0.59 ± 0.05 |  | 0.38 ± 0.03* |  | 0.62 ± 0.07 |  | 0.78 ± 0.07 |  | 0.98 ± 0.08 |  | 1.05 ± 0.07 |
| NDUFC2 | 1.02 ± 0.09 |  | 1.04 ± 0.11 |  | 1.22 ± 0.13 |  | 1.88 ± 0.24* |  | 1.9 ± 0.17 |  | 2.12 ± 0.21 |
| NDUFS2 | 0.73 ± 0.1 |  | 0.26 ± 0.02* |  | 0.69 ± 0.07 |  | 0.84 ± 0.08 |  | 1.04 ± 0.08 |  | 0.82 ± 0.07 |
| NDUFS4 | 0.43 ± 0.06 |  | 0.21 ± 0.01* |  | 0.57 ± 0.05 |  | 0.82 ± 0.08* |  | 0.97 ± 0.09 |  | 1.14 ± 0.07 |
| NDUFS5 | 0.3 ± 0.04 |  | 0.17 ± 0.01* |  | 0.34 ± 0.04 |  | 0.34 ± 0.03 |  | 0.66 ± 0.06 |  | 0.7 ± 0.07 |
| NDUFS6 | 0.18 ± 0.07 |  | 0.18 ± 0.07 |  | 0.15 ± 0.08 |  | 0.29 ± 0.12 |  | 0.43 ± 0.2 |  | 0.61 ± 0.23 |
| NDUFS7 | 0.78 ± 0.08 |  | 0.38 ± 0.03* |  | 0.66 ± 0.08 |  | 1.06 ± 0.13* |  | 1.31 ± 0.14 |  | 1.29 ± 0.12 |
| NDUFV1 | 0.74 ± 0.08 |  | 0.36 ± 0.04* |  | 0.68 ± 0.07 |  | 0.89 ± 0.08 |  | 1.27 ± 0.1 |  | 1.26 ± 0.07 |
| NDUFV2 | 0.55 ± 0.07 |  | 0.28 ± 0.03* |  | 0.6 ± 0.09 |  | 0.55 ± 0.06 |  | 1.27 ± 0.08 |  | 1.05 ± 0.06 |
| NDUFV3 | 0.37 ± 0.04 |  | 0.2 ± 0.01* |  | 0.3 ± 0.03 |  | 0.3 ± 0.05 |  | 0.59 ± 0.05 |  | 0.54 ± 0.03 |
| *NDUFAF2* | 0.15 ± 0.01 |  | 0.07 ± 0.01* |  | 0.08 ± 0.01 |  | 0.18 ± 0.01* |  | 0.12 ± 0.01 |  | 0.17 ± 0.01* |

**Supplemental Table S11**. Continued.

| Gene name^+^ | Liver | | |  | WSM | | |  | Heart | | |
| --- | --- | --- | --- | --- | --- | --- | --- | --- | --- | --- | --- |
|  | CTRL |  | Fasted |  | CTRL |  | Fasted |  | CTRL |  | Fasted |
| SDHA | 0.46 ± 0.04 |  | 0.22 ± 0.02* |  | 0.38 ± 0.04 |  | 0.41 ± 0.03 |  | 0.72 ± 0.05 |  | 0.82 ± 0.06 |
| SDHB | 0.2 ± 0.04 |  | 0.13 ± 0.01 |  | 0.18 ± 0.02 |  | 0.28 ± 0.04* |  | 0.31 ± 0.03 |  | 0.4 ± 0.03 |
| SDHC | 0.77 ± 0.04 |  | 0.35 ± 0.03* |  | 0.37 ± 0.04 |  | 0.56 ± 0.05* |  | 0.88 ± 0.09 |  | 1.03 ± 0.11 |
| SDHD | 1.01 ± 0.18 |  | 0.42 ± 0.05* |  | 0.31 ± 0.05 |  | 0.45 ± 0.04* |  | 0.74 ± 0.05 |  | 0.74 ± 0.06 |
| *SDHAF1* | 0.05 ± 0.00 |  | 0.04 ± 0.00 |  | 0.02 ± 0.00 |  | 0.03 ± 0.01 |  | 0.04 ± 0.00 |  | 0.05 ± 0.00* |
| *SDHAF2* | 0.27 ± 0.01 |  | 0.11 ± 0.01* |  | 0.08 ± 0.01 |  | 0.08 ± 0.01 |  | 0.11 ± 0.01 |  | 0.14 ± 0.01* |
| CYB | 43.23 ± 4.41 |  | 22.76 ± 2.64* |  | 23.59 ± 2.93 |  | 30.08 ± 3.21 |  | 35.21 ± 4.3 |  | 56.55 ± 2.22* |
| CYC | 0.08 ± 0.02 |  | 0.09 ± 0.02 |  | 1.74 ± 0.20 |  | 1.91 ± 0.31 |  | 4.32 ± 0.47 |  | 4.19 ± 0.21 |
| CYC1 | 1.33 ± 0.20 |  | 0.95 ± 0.08 |  | 1.69 ± 0.20 |  | 2.07 ± 0.18 |  | 4.3 ± 0.44 |  | 4.48 ± 0.42 |
| UQCRFS1 | 0.31 ± 0.05 |  | 0.18 ± 0.02* |  | 0.45 ± 0.04 |  | 0.63 ± 0.04* |  | 0.48 ± 0.04 |  | 0.48 ± 0.04 |
| UQCRC1 | 1.19 ± 0.16 |  | 0.76 ± 0.05* |  | 0.74 ± 0.11 |  | 1.46 ± 0.28* |  | 2.41 ± 0.20 |  | 2.7 ± 0.22 |
| UQCRC2 | 1.28 ± 0.17 |  | 0.65 ± 0.04* |  | 1.15 ± 0.10 |  | 2.25 ± 0.15* |  | 2.25 ± 0.14 |  | 2.17 ± 0.17 |
| UQCRH | 0.67 ± 0.08 |  | 0.44 ± 0.04* |  | 0.06 ± 0.01 |  | 0.09 ± 0.01* |  | 0.35 ± 0.03 |  | 0.4 ± 0.03 |
| UQCRB | 2.5 ± 0.35 |  | 1.41 ± 0.11* |  | 3.44 ± 0.4 |  | 4.16 ± 0.61 |  | 5.57 ± 0.44 |  | 5.42 ± 0.5 |
| UQCRQ | 3.16 ± 0.42 |  | 1.59 ± 0.1* |  | 2.84 ± 0.21 |  | 4.16 ± 0.41* |  | 4.20 ± 0.27 |  | 3.63 ± 0.31 |
| UQCR10 | 0.92 ± 0.06 |  | 0.36 ± 0.03* |  | 0.86 ± 0.09 |  | 1.45 ± 0.18* |  | 2.74 ± 0.31 |  | 2.27 ± 0.26 |
| UQCR11-A | 2.46 ± 0.09 |  | 1.7 ± 0.19* |  | 0.86 ± 0.11 |  | 1.57 ± 0.18 |  | 3.27 ± 0.41 |  | 4.95 ± 0.41 |
| UQCR11-B | 0.4 ± 0.03 |  | 0.25 ± 0.03* |  | 0.26 ± 0.03 |  | 0.25 ± 0.03 |  | 0.58 ± 0.04 |  | 0.57 ± 0.04 |
| *UQCC* | 0.14 ± 0.01 |  | 0.12 ± 0.01 |  | 0.29 ± 0.02 |  | 0.58 ± 0.04* |  | 0.34 ± 0.04 |  | 0.37 ± 0.03 |
| COXI | 29.86 ± 2.32 |  | 14.95 ± 1.83* |  | 16.91 ± 2.36 |  | 26.72 ± 4.83 |  | 36.79 ± 4.54 |  | 65.58 ± 3.76* |
| COXII | 15.99 ± 1.06 |  | 6.12 ± 0.88* |  | 6.96 ± 0.74 |  | 9.72 ± 1.4 |  | 13.77 ± 1.55 |  | 23.55 ± 1.46* |
| COXIII | 20.38 ± 1.74 |  | 8.09 ± 0.9* |  | 9.3 ± 1.1 |  | 9.9 ± 1.16 |  | 18.04 ± 1.99 |  | 32.43 ± 1.78* |
| NDUFA4 | 6.41 ± 0.24 |  | 3.91 ± 0.46* |  | 4.65 ± 0.42 |  | 5.96 ± 0.55* |  | 3.71 ± 0.12 |  | 6.55 ± 0.43* |
| COX4a | 3.23 ± 0.19 |  | 2.23 ± 0.21* |  | 0.71 ± 0.07 |  | 0.91 ± 0.13 |  | 4.62 ± 0.41 |  | 3.46 ± 0.32 |
| COX4b | 2.20 ± 0.1 |  | 1.04 ± 0.13* |  | 1.54 ± 0.15 |  | 1.37 ± 0.25 |  | 3.96 ± 0.47 |  | 4.82 ± 0.32 |
| COX5a1 | 0.10 ± 0.01 |  | 0.08 ± 0.01 |  | 0.14 ± 0.02 |  | 0.27 ± 0.04* |  | 0.27 ± 0.03 |  | 0.27 ± 0.01 |
| COX5a2 | 1.85 ± 0.17 |  | 0.82 ± 0.06* |  | 0.91 ± 0.13 |  | 1.73 ± 0.21* |  | 4.29 ± 0.56 |  | 3.85 ± 0.31 |
| COX5b2 | 0.28 ± 0.08 |  | 0.12 ± 0.01* |  | 0.09 ± 0.01 |  | 0.07 ± 0.01 |  | 0.32 ± 0.04 |  | 0.27 ± 0.03 |
| COX6a1 | 0.26 ± 0.05 |  | 0.17 ± 0.02 |  | 0.11 ± 0.01 |  | 0.09 ± 0.01 |  | 1.11 ± 0.10 |  | 0.97 ± 0.10 |
| COX6a2 | 2.68 ± 0.19 |  | 0.9 ± 0.05* |  | 2.24 ± 0.25 |  | 2.56 ± 0.37 |  | 3.76 ± 0.27 |  | 3.48 ± 0.22 |
| COX6b1a | 2.39 ± 0.26 |  | 1.33 ± 0.13* |  | 2.09 ± 0.15 |  | 2.6 ± 0.27 |  | 1.96 ± 0.21 |  | 2.75 ± 0.16* |
| COX6b1b | 3.19 ± 0.21 |  | 1.84 ± 0.16* |  | 0.31 ± 0.11 |  | 0.43 ± 0.09 |  | 3.67 ± 0.49 |  | 4.42 ± 0.30 |
| COX6c1 | 3.98 ± 0.38 |  | 1.53 ± 0.16* |  | 3.09 ± 0.30 |  | 4.32 ± 0.52 |  | 6.42 ± 0.73 |  | 5.78 ± 0.47 |
| COX7a1 | 1.27 ± 0.14 |  | 0.58 ± 0.06* |  | 1.27 ± 0.14 |  | 1.47 ± 0.16 |  | 2.66 ± 0.13 |  | 2.51 ± 0.17 |
| COX7a2 | 1.07 ± 0.23 |  | 0.46 ± 0.04* |  | 0.11 ± 0.02 |  | 0.13 ± 0.01 |  | 0.66 ± 0.08 |  | 0.56 ± 0.05 |
| COX7b | 8.97 ± 0.82 |  | 1.74 ± 0.11* |  | 2.43 ± 0.28 |  | 2.66 ± 0.34 |  | 5.64 ± 0.63 |  | 4.52 ± 0.52 |
| COX7c | 1.79 ± 0.23 |  | 0.69 ± 0.04* |  | 1.81 ± 0.2 |  | 2.29 ± 0.19 |  | 4.45 ± 0.45 |  | 4.06 ± 0.45 |
| COX8a | 0.19 ± 0.02 |  | 0.14 ± 0.01 |  | 0.11 ± 0.02 |  | 0.07 ± 0.01* |  | 0.78 ± 0.06 |  | 0.73 ± 0.03 |
| COX8b | 3.99 ± 0.33 |  | 1.4 ± 0.10* |  | 2.47 ± 0.23 |  | 3.56 ± 0.46* |  | 9.69 ± 1.17 |  | 9.96 ± 1.03 |
| *SCO1* | 0.05 ± 0.01 |  | 0.04 ± 0.00 |  | 0.02 ± 0.00 |  | 0.04 ± 0.01* |  | 0.04 ± 0.00 |  | 0.04 ± 0.00 |
| *SURF1* | 0.17 ± 0.01 |  | 0.11 ± 0.01* |  | 0.09 ± 0.01 |  | 0.17 ± 0.01* |  | 0.12 ± 0.01 |  | 0.16 ± 0.00* |
| *COX15* | 0.07 ± 0.01 |  | 0.05 ± 0.01 |  | 0.07 ± 0.01 |  | 0.10 ± 0.02 |  | 0.13 ± 0.02 |  | 0.20 ± 0.03 |

**Supplemental Table S11**. Continued.

| Gene name^+^ | Liver | | |  | WSM | | |  | Heart | | |
| --- | --- | --- | --- | --- | --- | --- | --- | --- | --- | --- | --- |
|  | CTRL |  | Fasted |  | CTRL |  | Fasted |  | CTRL |  | Fasted |
| ATP5A1 | 3.29 ± 0.48 |  | 1.58 ± 0.11* |  | 1.90 ± 0.25 |  | 2.94 ± 0.3* |  | 4.95 ± 0.5 |  | 3.69 ± 0.19 |
| ATP5B | 5.52 ± 0.77 |  | 1.73 ± 0.16* |  | 4.87 ± 0.61 |  | 5.79 ± 0.85 |  | 7.35 ± 0.82 |  | 5.34 ± 0.66 |
| ATP5C1 | 2.99 ± 0.32 |  | 1.10 ± 0.09* |  | 2.41 ± 0.19 |  | 3.20 ± 0.35 |  | 3.67 ± 0.38 |  | 3.97 ± 0.25 |
| ATP5D | 1.96 ± 0.21 |  | 1.09 ± 0.11* |  | 1.29 ± 0.11 |  | 1.72 ± 0.2 |  | 2.15 ± 0.21 |  | 2.07 ± 0.13 |
| ATP5E | 2.23 ± 0.16 |  | 0.89 ± 0.08* |  | 2.21 ± 0.29 |  | 2.71 ± 0.3 |  | 3.21 ± 0.26 |  | 3.53 ± 0.29 |
| ATP5F1 | 3.23 ± 0.36 |  | 1.63 ± 0.16* |  | 2.57 ± 0.22 |  | 3.11 ± 0.22 |  | 4.47 ± 0.39 |  | 5.22 ± 0.32 |
| ATP5G1 | 8.00 ± 1.18 |  | 3.04 ± 0.34* |  | 5.80 ± 0.53 |  | 5.49 ± 0.55 |  | 10.08 ± 0.81 |  | 9.21 ± 0.86 |
| ATP5I | 0.19 ± 0.01 |  | 0.10 ± 0.01* |  | 0.57 ± 0.06 |  | 0.63 ± 0.12 |  | 0.88 ± 0.08 |  | 0.76 ± 0.07 |
| ATP5J2 | 3.20 ± 0.38 |  | 1.21 ± 0.11* |  | 1.84 ± 0.21 |  | 1.84 ± 0.22 |  | 4.91 ± 0.52 |  | 4.16 ± 0.35 |
| ATP5L | 4.07 ± 0.23 |  | 2.56 ± 0.23* |  | 1.15 ± 0.17 |  | 1.54 ± 0.18 |  | 1.59 ± 0.14 |  | 1.96 ± 0.11 |
| ATP5O | 3.80 ± 0.35 |  | 1.33 ± 0.12* |  | 2.05 ± 0.41 |  | 3.19 ± 0.37* |  | 4.59 ± 0.45 |  | 4.66 ± 0.51 |
| OSCP | 0.004 ± 0.00 |  | 0.003 ± 0.00 |  | 0.003 ± 0.00 |  | 0.002 ± 0.00 |  | 0.006 ± 0.00 |  | 0.008 ± 0.00 |
| *ATPAF2* | 0.02 ± 0.00 |  | 0.02 ± 0.00 |  | 0.01 ± 0.00 |  | 0.02 ± 0.00 |  | 0.04 ± 0.00 |  | 0.04 ± 0.00 |

^+^ Gene identity determined through BLAST searches: Complex I: ND2, NADH-ubiquinone oxidoreductase chain 2; ND5, NADH-ubiquinone oxidoreductase chain 5; NDUFA1, NADH dehydrogenase [ubiquinone] 1 alpha subcomplex subunit 1; NDUFA2, NADH dehydrogenase [ubiquinone] 1 alpha subcomplex subunit 2; NDUFA3, NADH dehydrogenase [ubiquinone] 1 alpha subcomplex subunit 3; NDUFA4, NADH dehydrogenase [ubiquinone] 1 alpha subcomplex subunit 4; NDUFA4-like2, NADH dehydrogenase [ubiquinone] 1 alpha subcomplex subunit 4-like 2; NDUFA5, NADH dehydrogenase [ubiquinone] 1 alpha subcomplex subunit 5; NDUFA6, NADH dehydrogenase [ubiquinone] 1 alpha subcomplex subunit 6; NDUFA7, NADH dehydrogenase [ubiquinone] 1 alpha subcomplex subunit 7; NDUFA8, NADH dehydrogenase [ubiquinone] 1 alpha subcomplex subunit 8; NDUFA9, NADH dehydrogenase [ubiquinone] 1 alpha subcomplex subunit 9; NDUFA12, NADH dehydrogenase [ubiquinone] 1 alpha subcomplex subunit 12; NDUFB1, NADH dehydrogenase [ubiquinone] 1 beta subcomplex subunit 1; NDUFB2, NADH dehydrogenase [ubiquinone] 1 beta subcomplex subunit 2; NDUFB3, NADH dehydrogenase [ubiquinone] 1 beta subcomplex subunit 3; NDUFB4, NADH dehydrogenase [ubiquinone] 1 beta subcomplex subunit 4; NDUFB5, NADH dehydrogenase [ubiquinone] 1 beta subcomplex subunit 5; NDUFB6, NADH dehydrogenase [ubiquinone] 1 beta subcomplex subunit 6; NDUFB8, NADH dehydrogenase [ubiquinone] 1 beta subcomplex subunit 8; NDUFB9, NADH dehydrogenase [ubiquinone] 1 beta subcomplex subunit 9; NDUFB10, NADH dehydrogenase [ubiquinone] 1 beta subcomplex subunit 10; NDUFB11, NADH dehydrogenase [ubiquinone] 1 beta subcomplex subunit 11; NDUFC1, NADH dehydrogenase 1 subunit C1; NDUFC2, NADH dehydrogenase 1 subunit C2; NDUFS2, NADH dehydrogenase iron-sulfur protein 2; NDUFS4, NADH dehydrogenase iron-sulfur protein 4; NDUFS5, NADH dehydrogenase iron-sulfur protein 5; NDUFS6, NADH dehydrogenase iron-sulfur protein 6; NDUFS7, NADH dehydrogenase iron-sulfur protein 7; NDUFV1, NADH dehydrogenase [ubiquinone] flavoprotein 1; NDUFV2, NADH dehydrogenase [ubiquinone] flavoprotein 2; NDUFV3, NADH dehydrogenase [ubiquinone] flavoprotein 3; NDUFAF2, NADH dehydrogenase (ubiquinone) 1 alpha subcomplex, assembly factor 2; Complex II: SDHA, Succinate dehydrogenase [ubiquinone] flavoprotein subunit; SDHB, Succinate dehydrogenase [ubiquinone] iron-sulfur subunit; SDHC, Succinate dehydrogenase cytochrome b560 subunit; SDHD, Succinate dehydrogenase [ubiquinone] cytochrome b small subunit B; SDHAF1, Succinate dehydrogenase assembly factor 1; SDHAF2, Succinate dehydrogenase assembly factor 2; Complex III: Cytb, Cytochrome b; CYCS, Cytochrome c; Cyc1, Cytochrome c1, heme protein; UQCRFS1, Cytochrome b-c1 complex subunit Rieske; UQCRC1, Cytochrome b-c1 complex subunit 1; UQCRC2, Cytochrome b-c1 complex subunit 2; UQCRH, Cytochrome b-c1 complex subunit 6; UQCRB, Cytochrome b-c1 complex subunit 7; UQCRQ, Cytochrome b-c1 complex subunit 8; UQCR10, Cytochrome b-c1 complex subunit 9; UQCR11-A, Cytochrome b-c1 complex subunit 10 isoform A; UQCR11-B, Cytochrome b-c1 complex subunit 10 isoform B; UQCC, Ubiquinol-cytochrome c reductase complex chaperone CBP3 homolog; COXI, Cytochrome c oxidase subunit I; Complex IV : COXII, Cytochrome c oxidase subunit II; COXIII, Cytochrome c oxidase subunit III; COX4a, Cytochrome c oxidase subunit 4 isoform 1; COX4b, Cytochrome c oxidase subunit 4 isoform 2; COX5a1, Cytochrome c oxidase subunit 5A, mitochondrial-like isoform 1; COX5a2, Cytochrome c oxidase subunit 5A, mitochondrial-like isoform 2; COX5b2, Cytochrome c oxidase subunit 5B isoform 2; COX6a1, Cytochrome c oxidase subunit 6A isoform 1; COX6a2, Cytochrome c oxidase subunit 6A isoform 2; COX6b1a, Cytochrome c oxidase subunit VIb isoform 1a; COX6b1b, Cytochrome c oxidase subunit VIb isoform 1b; COX6c1, Cytochrome c oxidase subunit 6C-1; COX7a1, Cytochrome c oxidase subunit 7A1; COX7a2, Cytochrome c oxidase subunit 7A2; COX7b, Cytochrome c oxidase subunit 7B; COX7c, Cytochrome c oxidase subunit 7C; COX8a, Cytochrome c oxidase subunit 8A; COX8b, Cytochrome c oxidase subunit 8B; SCO1, SCO1 protein homolog; SURF1, Surfeit locus protein 1; COX15, Cytochrome c oxidase assembly protein COX15 homolog; Complex V : ATP5A1, ATP synthase subunit alpha; ATP5B, ATP synthase subunit beta; ATP5C1, ATP synthase subunit gamma; ATP5D, ATP synthase subunit delta; ATP5E, ATP synthase subunit épsilon; ATP5F1, ATP synthase subunit b; ATP5G1, ATP synthase lipid-binding protein; ATP5I, ATP synthase subunit e; ATP5J2, ATP synthase subunit f; ATP5L, ATP synthase subunit g; ATP5O, ATP synthase subunit O; OSCP, Protein OSCP1; ATPAF2, Mitochondrial F1 complex assembly factor 2.
